# Disulfide-compatible phage-assisted continuous evolution in the periplasmic space

**DOI:** 10.1038/s41467-021-26279-8

**Published:** 2021-10-13

**Authors:** Mary S. Morrison, Tina Wang, Aditya Raguram, Colin Hemez, David R. Liu

**Affiliations:** 1 Merkin Institute of Transformative Technologies in Health Care, Broad Institute of Harvard and MIT, Cambridge, MA 02142 USA; 2 Department of Chemistry and Chemical Biology, Harvard University, Cambridge, MA 02138 USA; 3 Howard Hughes Medical Institute, Harvard University, Cambridge, MA 02138 USA

**Keywords:** Genetic engineering, Protein design, Synthetic biology, Chemical biology

## Abstract

The directed evolution of antibodies has yielded important research tools and human therapeutics. The dependence of many antibodies on disulfide bonds for stability has limited the application of continuous evolution technologies to antibodies and other disulfide-containing proteins. Here we describe periplasmic phage-assisted continuous evolution (pPACE), a system for continuous evolution of protein-protein interactions in the disulfide-compatible environment of the *E. coli* periplasm. We first apply pPACE to rapidly evolve novel noncovalent and covalent interactions between subunits of homodimeric YibK protein and to correct a binding-defective mutant of the anti-GCN4 Ω-graft antibody. We develop an intein-mediated system to select for soluble periplasmic expression in pPACE, leading to an eight-fold increase in soluble expression of the Ω-graft antibody. Finally, we evolve disulfide-containing trastuzumab antibody variants with improved binding to a Her2-like peptide and improved soluble expression. Together, these results demonstrate that pPACE can rapidly optimize proteins containing disulfide bonds, broadening the applicability of continuous evolution.

## Abstract (web summary)

The directed evolution of antibodies yields important tools for research and therapy. Here the authors develop a periplasmic phage-assisted continuous evolution platform for improvement of protein-protein interactions in the disulfidecompatible E. coli periplasm.

## Introduction

Antibodies and their engineered derivatives are important treatments for diverse inflammatory, autoimmune, and infectious diseases, as well as many cancers, including HER2-positive breast cancer, non-Hodgkin’s lymphoma, and melanoma^1^. Monoclonal antibodies (mAbs) and their derivatives now represent the largest class of therapeutic protein drugs, with 82 therapeutic antibodies currently approved by the FDA and hundreds in clinical trials^2^.

Antibody-based therapies are limited by high development costs^1,3^. Directed evolution has the potential to decrease cost and accelerate the development of novel and potent antibodies. While multiple selection systems have been shown to evolve new antibody–antigen interactions in *E. coli*, including phage display^4–7^, APEx^8^, FLI-TRAP^9^, cyclonal^10^, BAD^11^, inner-membrane display^12^, and AHEAD^13^, many of these techniques require researcher intervention to carry out time-intensive steps. Continuous selection platforms, in which all stages of the evolutionary cycle are carried out by automated or in vivo processes without the need for researcher invention, have the potential to substantially streamline antibody development^4–27^.

**Fig. 1:**
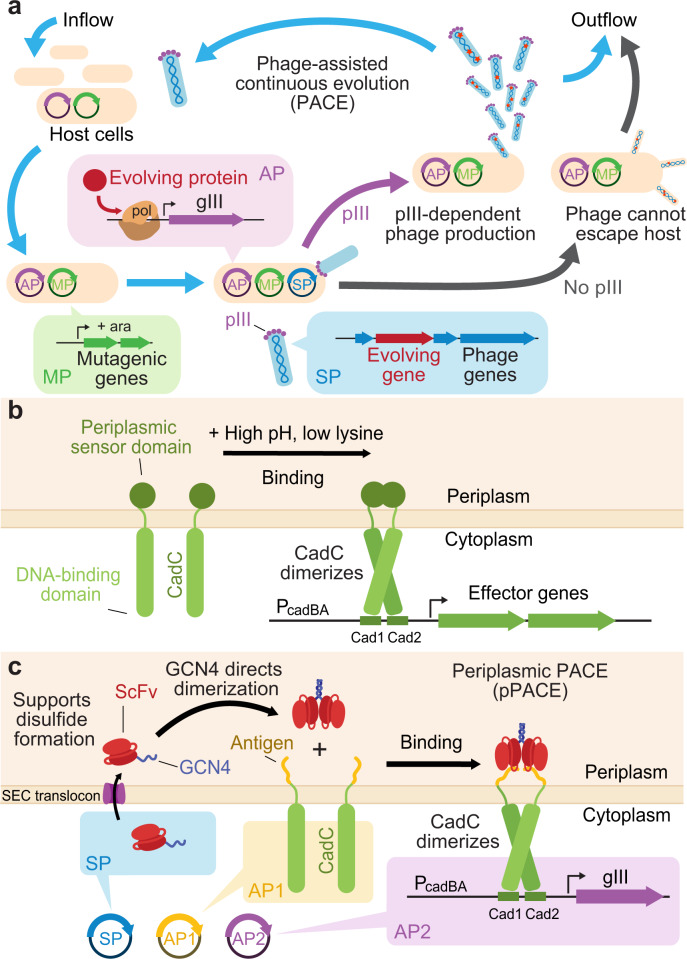
Periplasmic PACE (pPACE) selection system. **a** Overview of PACE. Selection phage (SP, blue) encode an evolving protein (red) in place of the native phage gene III (gIII), which encodes essential phage protein pIII. Host cells (tan) are transformed with a mutagenesis plasmid (MP, green) and one or more accessory plasmids (AP, purple) encoding selection-specific genes. The selection links the desired function of the evolving protein to expression of gene III. Induction of the MP with arabinose rapidly mutates the evolving gene. Phage encoding functional variants of the evolving protein trigger gIII transcription and pIII translation, and are thus able to propagate in a fixed-volume “lagoon”, while phage with nonfunctional variants are diluted out of the lagoon over time. **b** Native *E. coli* CadC signaling function^54,56^. The CadC sensory domain (green) dimerizes under conditions of high pH and low lysine in the periplasm, leading to dimerization of the cytoplasmic component of CadC and activation of P_cadBA_^54,56^. **c** Periplasmic PACE schematic. Phage encodes an evolving protein fused to a GCN4 leucine zipper. Following periplasmic export, GCN4 directs dimerization of the scFv−GCN4 species. Upon binding the target antigen (yellow), the dimeric evolving protein brings together two monomers of CadC linked to the antigen. Once in close proximity, the cytoplasmic DNA-binding domains of dimeric CadC cooperatively bind the DNA elements Cad1 and Cad2 of promoter P_cadBA_, inducing transcription of gIII and phage propagation.

Phage-assisted continuous evolution (PACE) is a rapid directed evolution system capable of evolving proteins over days or weeks, with minimal human intervention during evolution^28^. In PACE, an evolving protein is encoded in place of gene III (gIII) in the genome of M13 bacteriophage (Fig. 1a). An accessory plasmid (AP) within a host *E. coli* cell expresses gIII in response to the desired function of the evolving protein. As phage depends on pIII, the protein product of gIII, to efficiently infect host cells, PACE links the desired property of an evolving protein with phage fitness^28^. Phage propagates in a fixed-volume vessel (the “lagoon”) that is diluted with a constant inflow of new host cells from a population maintained in a chemostat. Phage that fails to propagate is quickly washed out of the lagoon by inflowing cells. Selection pressure is controlled by moderating the flow rate, and by modifying the genetic circuit governing gIII expression on the AP. Inducible mutagenesis plasmids (MPs) elevate the error rate of DNA replication when induced, allowing simultaneous selection and mutagenesis^28,29^. One complete generation of evolution occurs with each phage reproductive cycle (~10 min to 1 h)^28^.

PACE has been used to evolve diverse classes of proteins, including polymerases^19,30^, proteases^31–33^, tRNA synthetases^34^, agricultural toxins^28^, TALENs^35^, Cas9 variants^36,37^, dehydrogenases^38^, deaminase enzymes^39^, antibody fragments^39^, cytosine base editors^40^, adenine base editors^41^ and biosynthetic pathways^42^. However, continuous in vivo evolution platforms, including PACE, have thus far been limited to the cytoplasm of the host cell^19,23–28,30–42^. Confining selections to the cytoplasm maintains the linkage between genotype and phenotype. In both prokaryotes and eukaryotes, however, the cytoplasm is a chemically reducing environment, and does not support the formation of cysteine disulfide linkages. Disulfide bonds are crucial determinants of stability and proper folding for many proteins, including antibodies and antibody fragments. Loss of stabilizing disulfide bonds often leads to aggregation during cytoplasmic expression^43^, making disulfide-enriched proteins a challenging class of proteins to evolve by currently available continuous directed evolution techniques^11,39,44–49^.

Disulfide bond formation can be supported in the cytosol through the expression of thiol oxidase and disulfide isomerase in the cytoplasmic space^50^, but introducing non-native oxidative chemistry into the bacterial cytoplasm increases cellular stress, membrane impairment and aggregation^51^, a hurdle for the continuous-flow and liquid-handling devices used in continuous directed evolution. Alternatively, directed evolution can be applied to compensate for the loss of disulfides, but this process adds complexity and steps that are not ultimately necessary in proteins intended for use outside the cell. Compensatory stabilizing mutations may also result in trade-off costs to target affinity^39,45–49^, limiting the scope and relevance of the resulting proteins for use outside of cells. Finally, binding affinity evolutions in the cytoplasm are not compatible with disulfides in the target protein, excluding many extracellular antigens of therapeutic interest. It is thus more biologically relevant to evolve disulfide-containing proteins in oxidizing environments if the protein is intended for extracellular use.

The bacterial periplasm is an oxidizing environment that supports the formation of disulfides in proteins such as antibodies^43,52^. Expression of evolving proteins in the periplasm permits disulfide bond formation while retaining the evolving protein within the bacterial host cell.

In this study, we demonstrate a PACE system for the continuous evolution of proteins in the periplasmic space. This platform supports the formation of disulfide bonds in the evolving protein of interest and represents, to our knowledge, the first application of PACE to interactions occurring in a cellular compartment other than the cytoplasm and the first continuous in vivo evolution of proteins under oxidizing conditions. Periplasmic PACE (pPACE) can be tuned to select for enhanced soluble expression in addition to enhanced binding activity.

We validate pPACE by restoring binding in the homodimeric protein YibK and in the Ω-graft scFv. We apply pPACE to evolve a minimized form of the antibody-drug trastuzumab (Herceptin), achieving up to 2.5-fold improved binding of a Her2-mimetic peptide and 6-fold increased soluble expression, without any loss of native Her2 affinity. Together, these results establish pPACE as a method that substantially expands the scope of continuous protein evolution to include proteins that require a non-reducing environment to fold or function.

## Results

### CadC activates transcription upon periplasmic binding

A periplasmic protein-protein interaction selection system must convert a periplasmic binding event into a cytoplasmic transcriptional activation event. We examined transmembrane signaling proteins that physically link protein-protein binding in the periplasm with transcription in the cytoplasm. CadC is a native *E. coli* sensor protein and a member of the ToxR-like receptor family. Several members of the ToxR family have served as the basis for engineered sensors of periplasmic or transmembrane interactions^53–55^. CadC consists of a periplasmic sensor domain, a transmembrane helix, and a DNA-binding cytoplasmic domain (Fig. 1b). Under stress conditions, the periplasmic sensor domains from two CadC molecules homodimerize, bringing together the cytoplasmic DNA-binding domains to generate two cooperative DNA-binding sites, which then bind two DNA motifs, Cad1 and Cad2, on the CadBA promoter (P_cadBA_) to initiate gene transcription^54,56^. Replacement of the sensor domain with a dimerizing protein leads to constitutive activation of P_cadBA_^53^. CadC thus converts binding in the periplasm into cytoplasmic transcriptional activation.

**Fig. 2:**
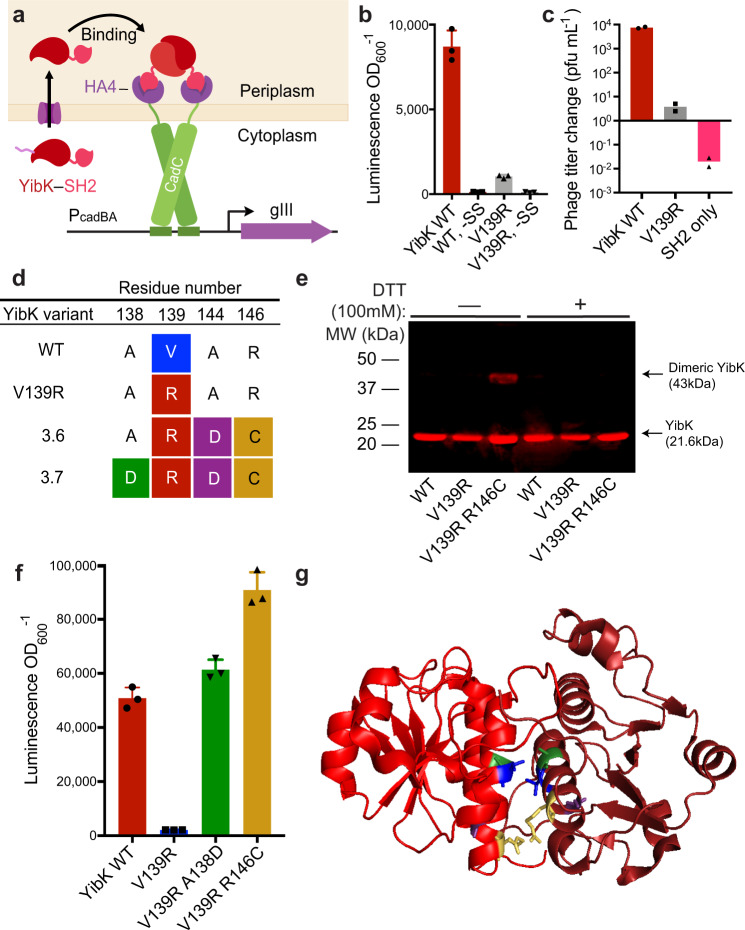
Periplasmic phage-assisted non-continuous evolution of the dimeric knottin YibK rescues binding mutants and evolves new disulfide bonds. **a** Schematic of homodimeric YibK selection. HA4 monobody (purple) recruits SH2 (pink) to CadC, and CadC monomers (green) are brought together by homodimerization of YibK (red). **b** Transcriptional activation assay comparing the performance of wild-type YibK−SH2 construct (WT) to the V139R binding mutant in the presence and absence of a signal sequence (SS) to direct periplasmic export. The architecture of the luciferase-based transcriptional reporter is shown in Supplementary Fig. 2a. Bar values and error bars represent the mean and s.d. of three independent biological replicates. **c** Phage propagation assay. Mid-log-phase cultures of selection strains were inoculated with phage and allowed to propagate overnight before determining titer. WT SS−YibK−SH2 phage enriches robustly, while the YibK V139R point mutant in the same construct enriches weakly and phage encoding only SP−SH2 fail to enrich. Bar values represent the mean of two independent biological replicate experiments carried out on separate days. (**d**) PANCE of YibK variant V139R evolves variants 3.6 and 3.7, showing two compensatory point mutations, A138D and R146C. R146C establishes a novel intermolecular disulfide bridge, resulting in a covalently bonded dimeric species that can be eliminated by the addition of a reducing agent, as shown by Western blot of purified YibK protein (**e**). The full gel image and corresponding Coomassie gel are provided in Supplementary Fig. 4b–c. The experiment was repeated once with similar results. **f**, **g** A138D restores wild-type activity in a V139R background in transcription assays (**f**), and likely forms a salt bridge with R139, as seen in the crystal structure of YibK dimer (**g**). Positions 138 (green) and 139 (blue) are in contact at the dimer interface. PDB ID = 1MXI^62^. Bar values and error bars in (**f**) represent the mean and s.d. of three biological replicates. Source data are provided as a Source Data file.

We reasoned that CadC could form the basis of a PACE selection for protein-protein binding in the periplasmic space (Fig. 1c). First, we optimized P_cadBA_ (Supplementary Fig. 1) and deleted the host genomic *cadCBA* operon to minimize background transcription (Supplementary Fig. 2). To validate that protein binding could trigger transcription at P_cadBA_, we expressed CadC with its sensory domain replaced by the HA4 monobody, a high-affinity monobody that binds the SH2 domain of ABL1 kinase^57^. We then expressed YibK, a homodimeric knottin protein, fused to the SH2 binding target of HA4. This construct was directed to the periplasm by an N-terminal signal sequence (SS) peptide derived from alkaline phosphatase A (PhoA),^58^. YibK homodimerization should trigger dimerization of the CadC–HA4 fusion via binding of HA4 to the SH2 domain fused to YibK, resulting in activation of P_cadBA_. Indeed, we observed that expression of SS–YibK–SH2 directed P_cadBA_ transcriptional activation 66-fold over expression of cytoplasmic YibK–SH2 as measured by transcription assay (Fig. 2b).

The point mutation V139R blocks YibK dimerization by disrupting hydrophobic interactions between YibK monomers and preventing a final folding transition to the native YibK structure^59,60^. The *K*_D_ values for dimerization of wild-type YibK and V139R YibK are <1 nM and 360 µM, respectively^60^. The introduction of V139R resulted in >8-fold loss of P_cadBA_-directed LuxAB expression (Fig. 2b), establishing that protein-protein affinity determines the degree of transcriptional activation at P_cadBA_.

To link binding in the periplasm to phage propagation, we drove gIII expression with P_cadBA_. We challenged phage encoding SS–YibK–SH2 to propagate in overnight culture on host cells expressing CadC–HA4 and P_cadBA_-driven gIII. Phage encoding wild-type YibK propagated more than three orders of magnitude more efficiently in this periplasmic PACE system than V139R YibK phage, demonstrating that pPACE links target protein binding in the periplasm to phage propagation through P_cadBA_ activation and production of pIII (Fig. 2c).

### Periplasmic phage-assisted evolution of YibK

To validate the pPACE selection for periplasmic protein-protein binding, we challenged the system to evolve homodimeric YibK variants starting from phage encoding the monomeric V139R variant. We adapted pPACE into the format of PANCE (phage-assisted non-continuous evolution)^37,38,41,61^, a non-continuous form of PACE in which host-phage populations undergo serial daily passaging in lieu of continuous flow, permitting a less stringent and more sensitive initial selection. After three PANCE passages, phage titers increased robustly (Supplementary Fig. 3b).

YibK variants evolved mutations that restore YibK dimerization. On the YibK dimer interface, V139 forms a hydrophobic contact with A138’ of its binding partner^62^. Evolving phage did not directly revert the V139R point mutation. However, in PANCE-evolved clone 3.7, residue A138 mutated to an aspartic acid (GCC to GAT), completely restoring affinity as measured by P_cadBA_ transcriptional activation (Fig. 2d, f). R146, which is in close proximity to R146’, was converted to a cysteine residue in seven of eight sequenced phage (CGT to TGT; Fig. 2g; Supplementary Fig. 3d), resulting in stronger transcriptional activation of P_cadBA_ than wild-type YibK. Remarkably, we found that R146C results in an intermolecular disulfide bridge. The covalently bound species can be seen by SDS-PAGE in purified YibK protein, as a ~43 kDa band representing the dimeric form of the 21.6 kDa monomer (Fig. 2e, Supplementary Fig. 4b, c). In whole-cell lysates, a ~60 kDa band representing the dimer of 30 kDa YibK–SH2 can also be visualized (Supplementary Fig. 4d-e). Both dimeric species are lost upon the addition of reducing agent. Together, these results establish that the periplasmic selection platform is capable of restoring and improving the stable homodimerization of a monomeric protein by multiple mechanisms, including the evolution of novel disulfide bridges.

### Periplasmic evolution of antibody–antigen affinity

Next, we applied pPACE to antibody–antigen binding. Full-length antibodies can be engineered into smaller forms such as single-chain variable fragments (scFvs), comprising only the heavy and light chain variable regions (V_H_ and V_L_) tethered by a flexible synthetic linker^1,2^. ScFvs are small (~30 kDa), can be produced in *E. coli*, exhibit improved tissue penetration, and can be readily conjugated to drug molecules, effector proteins and chimeric antigen receptors^1,3^, making them prime candidate molecules for directed evolution approaches. Heterologous expression of scFvs in *E. coli* typically involves export into the periplasm using an N-terminal signal sequence^3,63^.

**Fig. 3:**
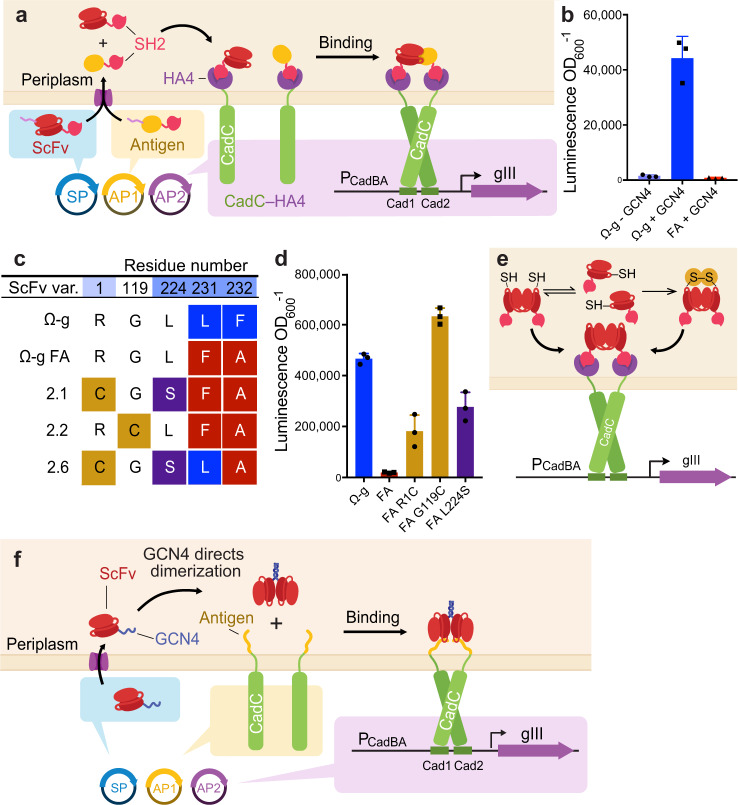
Initial design of pPACE and mechanism of selection survival through homodimerization. **a** Schematic overview of initial selection design. **b** Luminescence-based transcriptional activation assay comparing the performance of Ω-graft (abbreviated Ω-g) to the L231F F232A (here abbreviated FA) binding mutant in the presence and absence of its cognate antigen, a monomeric form of GCN4(7P14P) (abbreviated GCN4 in b) in the system diagrammed in (**a**). **c** PACE generates multiple variants with spontaneous N-terminal or 4X GGGS linker cysteine residues in addition to variants reversing mutation L231F. Full results can be found in Supplementary Fig. 5b. **d** Transcriptional activation assay. In a non-binding background, N-terminal cysteines drive partial or complete restoration of P_cadBA_ transcriptional activation, suggesting a mechanism of surviving the selection by the formation of novel disulfide bonds that generate covalent homodimeric scFvs, as shown in (**e**). Homodimeric scFv–SH2 fusions are able to bind drive CadC–HA4 dimerization without the involvement of the antigen. Bar values and error bars in (b) and (d) represent the mean and s.d. of three independent biological replicates. (**f**) Novel selection architecture designed to alleviate dimerization issues addressed above. A signal sequence (not depicted) directs export of scFv–GCN4 to the periplasmic space. The dimerizing leucine zipper GCN4 can be used to direct dimerization of scFvs in a predictable manner. Source data are provided as a Source Data file.

We challenged pPACE to evolve scFv antibodies. We chose the Ω-graft antibody scFv, which targets the leucine zipper GCN4 with *K*_d_ ~500 pM^39,64^. To determine whether an antibody–antigen interaction could drive CadC dimerization, we expressed CadC–HA4 and Ω-graft–SH2, with or without co-expression of a monomeric form of the leucine zipper GCN4^65^ (GCN4(7P14P)) fused to SH2. In this architecture, the binding of Ω-graft to GCN4 drives dimerization of CadC–HA4 bound to Ω-graft–SH2 and a CadC–HA4 molecule bound to GCN4–SH2, creating a four-part complex (Fig. 3a). The addition of GCN4 led to a 30-fold increase in P_cadBA_-driven LuxAB expression (Fig. 3b). In contrast, the substitution of a Ω-graft double point mutant L231F F232A, which impairs binding to GCN4 by >7,000-fold^64^, in place of wild-type Ω-graft led to a 55-fold decrease in transcriptional activation (Fig. 5b). Collectively, these results indicate that our selection can link scFv target binding to transcriptional activation of P_cadBA_.

To determine whether pPACE can distinguish between functional and nonfunctional forms of Ω-graft, we performed a competitive mock-selection experiment without mutagenesis. We seeded host cells expressing CadC–HA4 and GCN4–SH2 and encoding P_cadC_-driven gene III on the AP with a mixture of selection phage containing a 1:1,000 ratio of unmutated Ω-graft–SH2 selection phage to L231F F232A mutant–SH2 selection phage and carried out PACE and PANCE. Within 12 h of PACE or following two PANCE passages, unmutated Ω-graft variants dominated both populations, enriching ≥1000-fold (Supplementary Fig. 5a), demonstrating that the selection platform can be used to selectively propagate phage encoding a target-binding antibody scFv.

### Regulating scFv periplasmic export

In the small volume of the periplasm, minor changes in protein expression level have a large impact. An evolving SP might achieve increased fitness by modifying the promoter driving scFv expression to increase scFv levels and compensate for a poor *K*_D_. We reasoned that controlling scFv export to the periplasm would be desirable to maintain selection pressure. Further, regulating the level of periplasm-targeted scFv protein could drive two simultaneous selections: for high affinity to the target and for increased solubility of the scFv, to raise the effective concentration of scFv. We therefore adapted a key aspect of a related PACE selection, soluble expression PACE or SE-PACE^39^. SE-PACE uses a trans-splicing intein to reconstitute two fragments into a single functional protein, integrating transcription from two promoters into one output^39,66^. In SE-PACE, intein-mediated splicing reconstitutes the signal sequence peptide of pIII, which must enter the periplasmic space for phage to propagate^39^.

We split the PhoA-derived signal sequence (SS)^58^ into two halves, consisting of amino acids 1–8 and 9–21 (Supplementary Fig. 6). These two halves were fused, respectively, to the N- and C-terminal portions of the *Nostoc punctiforme* (Npu) trans-splicing DnaE intein^67^. SS amino acids 1–8 were fused to the N-terminal half of the Npu intein on a host AP1, ensuring that phages are not able to evolve the increased expression of this component. The C-terminal half of the Npu intein, fused to SS amino acids 9–20 and the evolving scFv, was encoded on the selection phage. Following translation of both fusion proteins, intein-mediated splicing reconstitute full-length SS–scFv, allowing periplasmic export (Supplementary Fig. 6).

Using the Ω-graft pPACE selection described above, we observed that expressing Ω-graft–SH2 with its SS split into two polypeptides, each fused to half of the Npu intein, led to robust phage propagation, indicating reconstitution of the SS. In contrast, when we omitted the C-terminal domain of Npu from the SS_9–20_–Ω-graft–SH2 construct, phage failed to propagate (Supplementary Fig. 6c). Similarly, the expression of the SS_1–8_–NpuN component is necessary for propagation (Supplementary Fig. 6d). We further found that by expressing SS_1–8_–NpuN under small-molecule induction in the presence of NpuC–SS_9–20_–Ω-graft (34.8 kDa), we could drive periplasmic expression of Ω-graft scFv (30.2 kDa) in a dose-dependent manner (Supplementary Fig. 6g, h). This demonstrates that Npu can regulate the reconstitution of full-length SS for periplasmic export of scFvs and P_cadBA_ activation.

Under this intein-regulated system, the total amount of scFv exported to the periplasm, and thus available direct CadC dimerization, is limited by the availability of the intein-SS fragment encoded on the host AP. The researcher can modify the expression level of intein–SS_1–8_ fragment to limit the reconstitution of SS–scFv, and thus the amount of scFv exported to the periplasm, creating selection pressure for efficient expression of soluble scFv.

### Evolution of Ω-graft and overcoming scFv homodimerization

Next, we challenged pPACE to correct the L231F F232A binding mutation in the Ω-graft antibody scFv, using both a full-length N-terminal SS sequence and the intein-SS strategy described above, in order to select for affinity alone or affinity and soluble periplasmic expression. We aimed to apply pPACE to restore binding to GCN4 by correcting mutation L231F F232A.

PACE experiments using our original selection architecture (Fig. 3a) resulted in two genotypic outcomes. First, close to half of phage reverted mutation L231F to the wild type within 96 h of pPACE. Second, scFv variants developed cysteine residues at their N-termini or within the 4X GGGS linker connecting scFv VH and VL domains (Fig. 3c). Linker cysteines in particular appeared mutually exclusive to the desired L231F reversion (Supplementary Fig. 5b). We found that at both positions, a cysteine substitution resulted in higher transcriptional activation than reversion of position 231 to Leu (Fig. 3d). The insertion of a C-terminal Cys residue has been used to manufacture stable dimeric scFvs through the formation of a covalent disulfide^68^. We reasoned that an N-terminal or linker Cys residue might form a similar covalent linkage, generating stably homodimeric scFv–SH2. This homodimer circumvents the target-binding selection by binding two CadC–HA4 molecules and bring them into close proximity, without the involvement of the antigen (Fig. 3e).

**Fig. 4:**
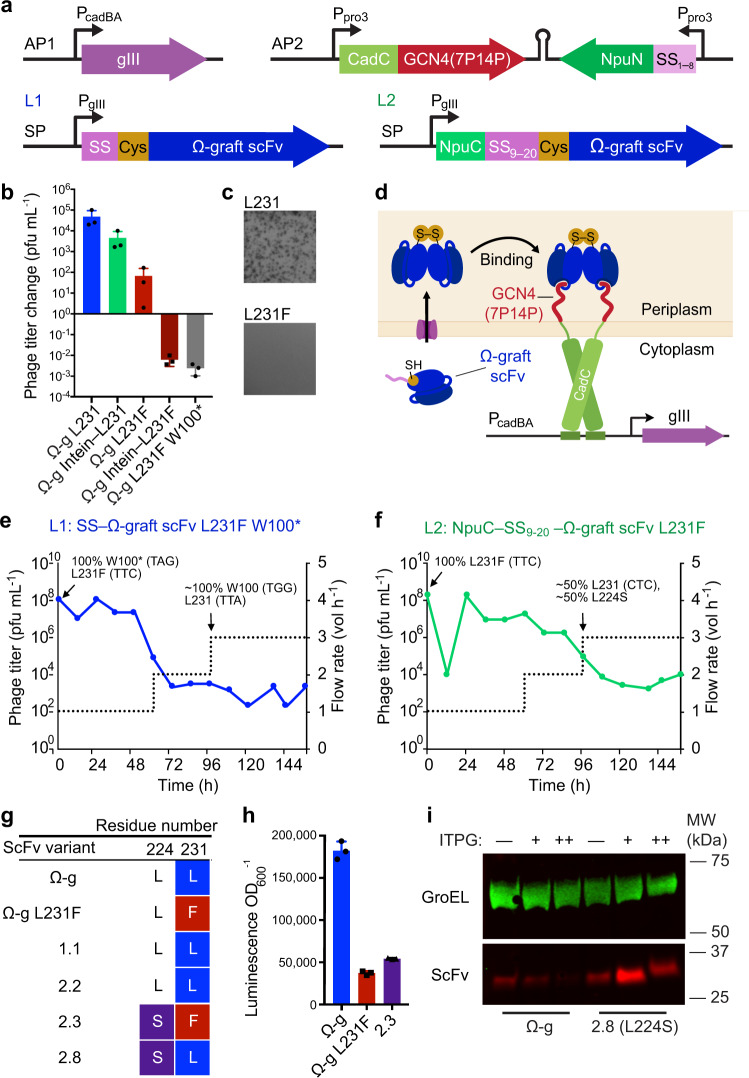
Second-generation pPACE selection reverts a binding mutant in Ω-graft scFv. **a** Schematic of periplasmic PACE architecture 2 components. Ω-graft (Ω-g) scFvs form covalent dimers through N-terminal cysteine residues. GCN4 monomeric variant 7P14P is used to avoid background dimerization of CadC. Promoter P_pro3_ is a low-level constitutive promoter^39^. P_gIII_ is a native phage promoter. **b** Overnight phage propagation assay of Ω-graft scFv variant SP, illustrating the effect of L231F on phage propagation. Introduction of a stop codon into position 100 of scFv (L231F−STOP) prevents phage propagation. Splitting the signal sequence using an intein (intein−L231/F) leads to reduced propagation. Bar values and error bars represent mean and s.d. of three biological replicates conducted on separate days. **c** Plaque assay visualizing overnight expansion of intein–SS_9−20_ phage variants L231 and L231F as in (**b**). Full plates provided in Supplementary Fig. 8c, d. **d** Ω-graft selection overview. After periplasmic export and SS cleavage, a cysteine is exposed at the N-terminus to mediate covalent disulfide bonding of two scFv monomers as described in Fig. 3e. Binding of GCN4 by dimeric scFvs leads to activation of P_cadBA_. **e**, **f** PACE was carried out over 156 h using full-length SS–scFv phage (**e**) or split-intein SS–scFv phage (**f**). To impose additional challenges, full-length SS–scFv phage were also challenged to correct a nonsense mutation. By 96 h, phage had converged upon solutions shown in (**g**) and in Supplementary Fig. 9a, b. Duplicates of each PACE experiment were evolved with similar outcomes, correcting W100* and enriching F231L in the replicate of (**e**) and discovering L224S and F231L in replicate of (**f**). **h** Luminescence assay shows increased P_cadBA_ activation as a result of L224S in an L231F background. Bar values and error bars represent mean and s.d. of three biological replicates. **i** Western blot showing Ω-graft and L224S evolved mutant, expressed from P_T7Lac_ in BL21*DE3 cells. L224S increases soluble Ω-graft scFv expression by roughly 8-fold. This experiment was repeated in biological triplicate on separate days with similar results. Full gel and densitometry analyses are provided in Supplementary Figs. 9c, d and 15g and in the Source Data file.

**Fig. 5:**
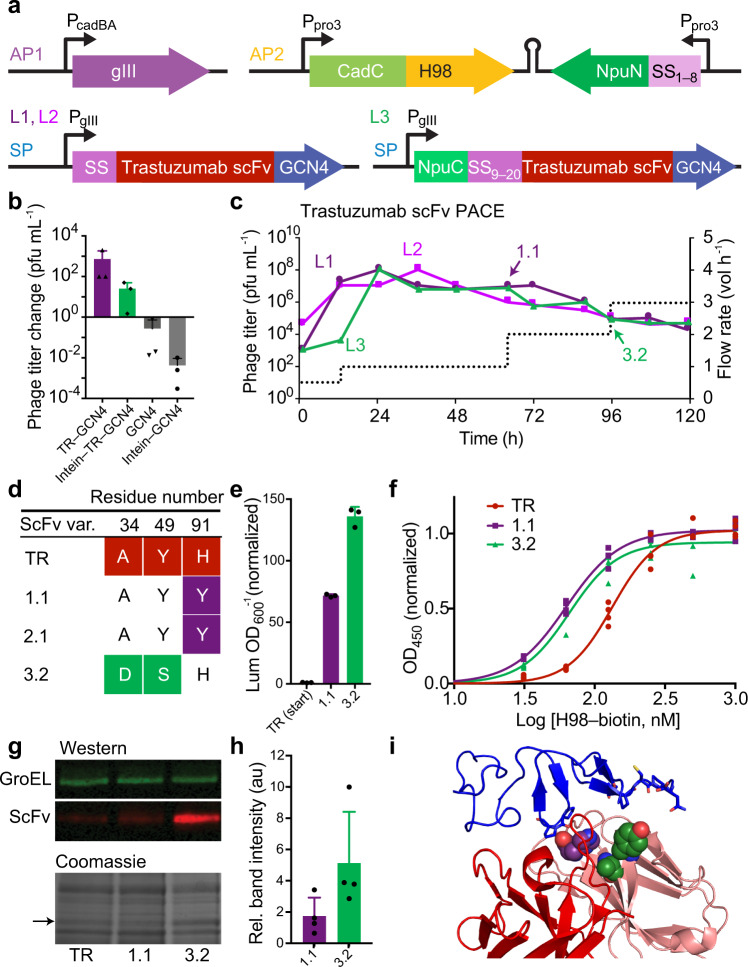
Evolution of trastuzumab variants with improved binding to a Her2-mimetic peptide. **a** Components of the second-generation periplasmic PACE system to evolve trastuzumab. The H98 peptide is a structural homologue of the Her2 epitope. A C-terminal dimeric GCN4 peptide directs dimerization of scFvs. **b** Phage propagation assay of starting genotypes and negative controls. Sequences with intein-split SS are indicated as ‘intein’. **c** PACE was carried out over 120 h using full-length (lagoons L1−L2, purple) or split-intein signal sequence (lagoon L3, green). By 96 h, all three lagoons converged on discrete solutions, shown in (**d**) and in Supplementary Fig. 10a. **e** Luminescence assay with trastuzumab (abbreviated TR, red) and evolved trastuzumab variants demonstrates increased P_cadBA_ activation. Luminescence/OD_600_ values are shown relative to that of trastuzumab. **f** ELISA shows modest improvement in binding. Values represent the mean and individual data points of four technical replicates from the same protein preparation. Data points at far ends of the binding curve, used to verify top and bottom values, can be found in Supplementary Fig. 10c. This experiment was repeated with four separate protein preparations and gave similar results. Average EC_50_ and Hill slope values from all replicate experiments can be found in Table 1. PAGE analysis of purified protein used in this representative ELISA is shown in Supplementary Fig. 16b. **g**, **h** Western blot and Coomassie-stained gels of TR and evolved variants expressed from the T_7Lac_ promoter in BL21*DE3 cells, showing improved soluble expression of variant 3.2. Full gels are shown in Supplementary Fig. 15a, b. Densitometry data reflects mean and s.d. of Western blot method and includes four independent biological replicates conducted on separate days. **i** Location of individual evolved mutations from PACE in the crystal structure of trastuzumab Fab bound to Her2 (PDB ID: 1N8Z^75^). Mutations are colored by lagoon origin as in (**c**). Bar values and error bars in (**b**), (**e**), and (**h**) represent the mean and s.d. of three independent biological replicates. Source data are provided as a Source Data file.

To prevent circumvention of the target-binding selection, we modified the selection architecture by fusing the GCN4(7P14P) antigen directly to CadC in place of HA4, to eliminate the possibility of scFv homodimerization resulting in selection survival (Fig. 3f). We created obligate homodimeric scFvs by removing the now-redundant SH2 domain fusion and either pre-installing an N-terminal cysteine in the Ω-graft scFv (Fig. 4a), or, as a more general strategy, by fusing a homodimerizing GCN4 leucine zipper domain C-terminal to the scFv (Fig. 3f; Fig. 5a, Supplementary Fig. 7). This strategy ensures that efficient dimerization does not depend on the properties of the scFv being evolved, since different scFvs homodimerize at different rates^69^. In this second-generation selection architecture, a dimeric scFv antibody must bind two CadC-fused antigens to activate P_cadBA_. Transcriptional assays suggested that mutation L231F accounts for the loss of binding, and that F232A alone has little effect (Supplementary Fig. 8e). We therefore considered reversion of F232A to be unnecessary in desired selection outcomes.

Using this second-generation architecture, phage encoding Ω-graft showed three orders of magnitude higher levels of propagation in overnight enrichment assays than phage encoding Ω-graft L231F (Fig. 4b, c). Incorporation of a nonsense mutation at position 100 (W100*) also led to strong de-enrichment of phage (Fig. 4b).

We challenged pPACE using the second-generation architecture to correct a stop codon at W100 in addition to the L231F binding defect mutation. Within 96 h of pPACE, phage reverted mutations correcting both deleterious mutations in population 1 (Fig. 4e, Supplementary Fig. 9a). In population 2, we used the split-intein signal sequence strategy described above to regulate periplasmic scFv expression in host cells (Fig. 4a, f). Due to the decreased fitness of intein-SS phage compared to phage with full-length SS (Fig. 4b), we did not challenge population 2 to correct a stop codon. Mutation F231L was present in ~50% of this population by 96 h and dominated the population by 156 h (Supplementary Fig. 9b). Phage in different populations accessed leucine codons at position 231 via two distinct point mutations, converting TTC to TTA or CTC (Fig. 4e, f). Importantly, we observed no new cysteines arising during evolution (Fig. 4g; Supplementary Fig. 9a). These results suggest that the second-generation pPACE selection prevents phage from passing the selection by evolving stable scFv–SH2 homodimers alone, and requires a tight scFv-antigen interaction in order to activate P_cadBA_.

In population 2, which used the intein-SS strategy, two point mutations, F231L and L224S, enriched as separate solutions present at a similar frequency at 96 h. Mutations F231L and L224S were observed in the same variant by 112 h (Supplementary Fig. 9b). Mutation L224V was previously reported to enhance cytoplasmic solubility of the Ω-graft scFv^39^. We compared the soluble expression of Ω-graft scFv and variant L224S with an N-terminal PhoA SS from the promoter P_T7Lac_ in BL21*DE3 cells by western blotting. We found that L224S increased soluble expression of Ω-graft by roughly 8-fold (Supplementary Fig. 9c, d).

Together, these findings demonstrate that pPACE can restore affinity of an antibody to an antigen, and that regulating periplasmic export of the evolving species using a split-intein signal sequence can support the evolution of improved soluble expression and improved binding. These results also show that the second-generation pPACE system avoids outcomes that circumvent the selection by homodimerizing the evolving protein, rather than by binding the target.

### Periplasmic PACE of novel trastuzumab scFv variants

We used the second-generation pPACE selection to evolve an scFv form of the antibody trastuzumab to bind a new target antigen. Trastuzumab targets the oncogenic receptor Her2 and is a successful first-line treatment for Her2^+^ breast cancers. Most trastuzumab-responsive tumors develop resistance to the drug within one year^70^. Second-line treatments can overcome resistance using multi-specific engineered antibodies, which combine variable domains of two or more mAbs to simultaneously target several epitopes, such as Her3, EGFR and VEGF kinase receptors^71–73^. The ability of pPACE to rapidly evolve affinity to novel epitopes could further broaden the targeting capacity of engineered multi-specific antibodies.

The Her2 mimetic peptide H98 was identified in a peptide library screen for trastuzumab binding, and bears structural similarity but no sequence homology to Her2^74^. We sought to apply pPACE to evolve an scFv form of trastuzumab with a higher affinity for the H98 peptide. We evolved trastuzumab scFv in the second-generation pPACE selection using either full-length SS or the split-intein SS strategy, resulting in mutually exclusive outcomes within 96 h of evolution. The H98 antigen was presented as a CadC–H98 fusion driven by a weak constitutive promoter on the AP. Trastuzumab was expressed as an scFv–GCN4 fusion to ensure dimerization, as we found that the use of a larger domain such as YibK to direct dimerization resulted in poor phage propagation (Fig. 5b), possibly due to excessive crowding of the periplasmic space.

Phages were allowed a 24-h period of evolutionary drift when pIII was provided freely with elevated mutagenesis^30^, to generate a large and diverse phage library. Phages were then subjected to high-stringency pPACE at increasing flow rates until titers plateaued (Fig. 5c, Supplementary Fig. 10). In populations 1 and 2, phage encoded the full-length SS. Both populations converged on a single point mutation, H91Y (variant 1.1, Fig. 5d). In population 3, periplasmic export was restricted through the split-intein strategy described above, leading to enrichment of a single variant (3.2) with mutations A34D Y49S. Periplasmic PACE experiments carried out at further increased stringencies did not result in the enrichment of any additional point mutations in the scFv (Supplementary Fig. 11).

Computational modeling indicates that trastuzumab interacts with H98 through heavy chain residues V33, R50, and Y105, and light chain residues T94 and N30^74^. In the trastuzumab crystal structure, residue T94 is proximal to residue H91 (H91Y in variant 1.1), and residue N30 is proximal to residue A34 (A34D in 3.2) (Supplementary Fig. 10e). Light chain residue Y49 is adjacent to residue A34 in a β-sheet, and mutation Y49S (variant 3.2) may help to accommodate the substitution of alanine for a bulky, charged aspartic acid at position 34 (PDB ID: 1N8Z^75^).

Trastuzumab and evolved variants show a similar, characteristic change in mobility consistent with the reduction of disulfides during SDS-PAGE under reducing conditions, suggesting that intra-chain disulfides are retained in evolved variants (Supplementary Fig. 12a). To examine the role of intra-chain disulfides in the stability and binding of trastuzumab variants, we abrogated the possibility of disulfide formation by replacing the disulfide-forming cysteine residues with serines. In the absence of disulfide bonds, both trastuzumab and evolved variants failed to induce transcription from P_cadBA_ (Supplementary Fig. 12b). These results further suggest that trastuzumab binding is likely dependent on intra-chain disulfides, in agreement with the findings of Wörn and Plückthun that expression of trastuzumab scFv without disulfide bonds results in insoluble protein^76^, and that these disulfides are preserved through pPACE. To ensure that the accumulation of insoluble cytoplasmic scFv does not impair host cell fitness, we carried out a growth timecourse, and found that scFv expression, with or without split-intein SS, had little to no effect on host cell growth (Supplementary Fig. 13).

Evolved variant 1.1 showed ~2.5-fold improved binding to H98 as measured by ELISA and little change in soluble expression (Fig. 5f-h, Table 1, Supplementary Fig. 10c, d). Evolved variant 3.2 was selected using the split-intein SS selection and showed a ~2-fold increase in affinity (Fig. 5f-h, Table 1, Supplementary Fig. 10c, d). To support ELISA data, we also carried out microscale thermophoresis (MST) (Supplementary Fig. 14, Supplementary Table 5). We note that in MST experiments, the upper bound of the binding curve was not accessible due to solubility limits of the H98 peptide in aqueous buffer (Supplementary Fig. 14), which may affect the accuracy of K_d_ determination. However, EC_50_ values determined by MST and ELISA reflect similar fold improvements for evolved variants over the starting scFv.

**Table 1:** Properties of trastuzumab scFv and evolved variants.

	EC_50_ (nM)^a^	Hill slope^a^	*T*_M_ (C°)
TR	160.7 ± 26.2	3.1 ± 0.6	68.5
1.1	63.6 ± 3.0	2.6 ± 1.4	72.5
3.2	77.6 ± 15.0	3.0 ± 1.0	62.5

Trastuzumab is abbreviated as TR. Values were determined by pooling means from four ELISA experiments conducted with separate protein preps, each with four technical replicates per ELISA experiment, and calculating mean and s.d. of pooled means. Melting temperature data reflects mean of two experiments conducted with separate protein preps, each consisting of four technical replicates. Source data are provided as a Source Data file.

Variant 3.2 showed substantial increases in soluble periplasmic expression (~5-fold as measured by western blotting and 2.5-fold as measured by Coomassie staining; see Supplementary Fig. 15), suggesting that restricting the level of scFv export to the periplasm selected for enhanced solubility. Evolved variants showed unchanged binding to Her2 in ELISA compared to that of trastuzumab scFv (Supplementary Fig. 10b). Evolved variants showed relatively unchanged thermal stability. Unevolved trastuzumab scFv had a melting temperature of 68.5 °C. We observed T_M_ increase of +4.0 ° for variant 1.1 and a T_M_ decrease of –5 °C for variant 3.2 (Supplementary Fig. 15f, Table 1).

## Discussion

Continuous directed evolution has the potential to significantly streamline the development of antibodies, but disulfide-containing proteins represent a significant challenge for current continuous evolution methods, which occur in the reducing cytoplasmic space. We developed a method for the continuous evolution of protein binding that takes place in the bacterial periplasm. Periplasmic PACE can rapidly generate proteins with improved binding and expression properties from a starting gene within several days of evolution. While the packaging limit of the M13 bacteriophage (12 kb) likely makes the evolution of full-length immunoglobulin (IgG) antibodies unwieldy, pPACE focuses evolution to the domains containing the complement-determining regions, maximizing the likelihood of discovering mutations that improve binding without increasing immunogenicity^77^. ScFv can then be grafted into scaffolds to produce full-length IgGs. Periplasmic PACE supports native disulfide bonds, which can be critical for the folding and stability of scFvs and other proteins in both prokaryotic and eukaryotic contexts^78^. Splitting the signal sequence into two halves, with one half expressed at a controlled level on a host plasmid, allows the researcher to define the extent of export to the periplasm, further modulating selection stringency.

We applied pPACE to evolve YibK variants with restored binding via two novel mechanisms in only three serial passages, Ω-graft antibody variants with restored binding and 8-fold improved solubility within 96 h of pPACE, and trastuzumab variants with up to 5-fold improved solubility and 2.5-fold improved binding affinity to a peptide antigen within 96 h of pPACE. Taken together, these studies establish that pPACE can evolve improved binding and expression profiles of antibodies and other proteins in the periplasmic space on short timescales.

In an oxidizing environment such as the extracellular space, intra-chain disulfides are highly conserved, and can make the Δ*G* of folding more favorable by 4–5 kcal mol^−1^, corresponding to an increase in folded states over unfolded states of roughly three orders of magnitude^78^. For non-intrabody applications such as CAR-T therapy, engineering disulfide-free scFvs is generally not desirable or necessary. Periplasmic PACE therefore offers a complementary strategy to other intracellular evolution methods by enabling continuous evolution for binding activity and soluble expression while conserving native disulfide linkages.

The properties of the periplasm offer opportunities that pPACE is well-suited to exploit. Protein channels in the outer membrane of *E. coli* render the periplasm permeable to water, ions, and hydrophilic solutes up to ~600 Da in size^79^. Further, the pH of the periplasm mirrors the pH of the extracellular environment^80^. The composition of the growth medium used in pPACE may strongly influence the folding and activity of evolving proteins. We speculate that pPACE might be used in the evolution of proteins with unusual pH requirements, and could be leveraged for applications involving small-molecule substrates.

Due to the innate constrains of *E. coli* expression and the architectural requirements of the CadC selection, periplasmic PACE is appropriate for the evolution of binding towards antigens that are small and well-behaved in the E. coli periplasm, do not contain essential glycosylations, and do not homodimerize. Large mammalian cell-surface antigens are not likely to behave well in pPACE, while peptides, very small proteins, and bacterial proteins are more likely to be compatible with pPACE. In this work, we have shown evolution towards peptide antigens (e.g, GCN4, 0.4 kDa) and YibK (21.6 kDa). First-generation architecture is appropriate for use with monomeric evolving proteins, while second-generation pPACE is appropriate for dimeric evolving proteins and antigens that can tolerate an N-terminal fusion.

ScFv phage with split-intein signal sequence propagated less robustly than their full-length SP counterparts (Figs. 4b, 5b). Under intein-regulated conditions only, both Ω-graft and trastuzumab phage evolved improved soluble periplasmic expression. Limiting the rate of periplasmic export appears to impose selection pressure for both soluble periplasmic expression and affinity. In a regime limited by the availability of the NpuN–SS_1-8_ fragment, mutations that improve overall expression levels are unlikely to have a large effect, as excess intein–SS_9-20_–scFv construct simply accumulates in cytoplasm. Notably, we did not observe increased scFv in the insoluble pellet fraction following intein-regulated pPACE (Supplementary Figs. 9d and 15c, d). We speculate that splitting the signal sequence selects for variants that limit aggregation or degradation occurring after intein-mediated splicing, mediate rapid periplasmic export, or facilitate folding in the periplasm.

High-micromolar and low-nanomolar *K*_D_ variants of YibK and Ω-graft performed very differently in pPACE. YibK variant 3.7 and Ω-graft variant 2.8 evolved beneficial mutations in addition to the mutation expected to restore binding affinity to low-nanomolar *K*_D_ levels. In the case of trastuzumab and the Her2 mimetic peptide H98, however, only modest affinity improvements were evolved from an initially moderate *K*_D_ (the *K*_D_ of trastuzumab IgG-H98 interaction is reported to be 1.4 μM^81^). This outcome may reflect the limited surface area of H98 available for molecular interaction, or may suggest stringency limitations in the selection. Consistent with these possibilities, further decreasing expression of antigen and pIII did not lead to enrichment of new trastuzumab genotypes (Supplementary Fig. 11). Indeed, mutations A34D and Y49S were not observed after high-stringency evolution, suggesting that expression increases may saturate the limited available H98 in this selection regime. We speculate that stringency might be further elevated by providing a competitive binder at the late stages of the selection.

We have shown that periplasmic PACE can improve both affinity and solubility of Ω-graft and trastuzumab scFvs, and can generate variants of homodimeric YibK with non-covalent and covalent linkages between subunits. To our knowledge, periplasmic PACE represents the first PACE selection for function in a cell compartment other than the cytoplasm, and the first continuous selection in the periplasmic space. We anticipate this system may be particularly useful for rapid optimization of binding and solubility, especially when evolving antibodies to engage antigens that contain disulfide bonds and are incompatible with cytoplasmic PACE.

## Methods

Nuclease-free water (Qiagen) was used for PCR reactions and cloning. PCR reactions were carried out using Phusion U Hot Start DNA polymerase (Thermo Fisher Scientific). Plasmids and SPs were cloned by USER assembly according to the manufacturer’s instructions^28^. For antibodies and antigens used in this work, synthesized gBlock gene fragments were obtained from Integrated DNA Technologies. *E. coli* native genes were amplified directly from genomic DNA. Plasmids were cloned and amplified using Turbo (New England BioLabs) cells. Plasmid DNA was amplified for sequencing purposes using the Illustra Templiphi 100 Amplification Kit (GE Healthcare Life Sciences); SPs were amplified by PCR using primers AB1793 and AB1396. Phages were sequenced using primers AR007, MM1081, MM1082, TW629, and TW1243. All primer sequences can be found in Supplementary Table 4. Sanger sequencing was used to confirm all plasmid sequences and to characterize SPs. Phage cloning and phage titer determination was carried out in strain S2208^28^. Clone-specific sanger sequencing data for phages sequenced can be found in the Source data spreadsheet.

Plasmids and phage used in this work can be found in Supplementary Tables 1–3. Antibiotic (Gold Biotechnology) working concentrations were as follows: carbenicillin 50 μg/mL, spectinomycin 50 μg/mL, chloramphenicol 25 μg/mL, kanamycin 50 μg/mL, tetracycline 10 μg/mL, streptomycin 50 μg/mL.

### Preparation and transformation of competent cells

To prepare chemically competent cells of strains S536, S1367 and S2208, overnight cultures were grown from single colonies and diluted 500-fold into 10 mL of 2xYT media (United States Biologicals) supplemented with appropriate antibiotics. Cells were grown at 37 °C with 230 RPM shaking to OD_600_ = 0.4–0.6 and pelleted by centrifugation at 4000*g* for 10 min at 4 °C. The cell pellet was then resuspended in 500 µL TSS (LB media supplemented with 2.5% v/v DMSO, 5% w/v PEG 3350, and 10 mM MgCl_2_). For transformations, 50 μL of competent cells were added to 1 µL plasmid in 50 μL pre-chilled KCM (100 mM KCl, 30 mM CaCl_2_, and 50 mM MgCl_2_ in H_2_O), incubated on ice for 15 min, heat shocked at 42 °C for 90 s and incubated on ice 2 min prior to recovery.

To prepare electrocompetent cells of strains S1021, S536, S1367, single colonies or glycerol stocks were grown up overnight and diluted 500-fold in 2xYT plus appropriate antibiotics. 10 mL of cells at OD_600_ 0.3-0.4 were pelleted by centrifugation at 4000*g* for 10 min at 4 °C. The cell pellet was resuspended in 1 mL ice-cold 10% glycerol and washed 3X with 1 mL ice-cold glycerol, pelleting at 10,000*g* for 1 min at 4 °C between washes and maintaining cells on ice between spins. The pellet was resuspended in 500 µL ice-cold 10% glycerol and the resulting mixture used fresh or else stored at −80 °C. For transformation, 1 µL each of up to three plasmids was added directly to 50 µL of electrocompetent cells prior to electroporation in pre-chilled cuvettes (Bio-Rad).

Cells were recovered for 1 h at 37 °C with shaking at 230 RPM in 1 mL of SOC media (New England BioLabs) and streaked on 2xYT media + 1.5% agar (United States Biologicals) plates containing the appropriate antibiotics before incubation at 37 °C for 12–18 h.

### *E. coli* strains

All luminescence assays and evolution experiments were carried out in *E. coli* strains S536 and S1367. These strains were engineered from PACE strains S1030^30^ and S2060^35^, respectively, using Lambda Red recombineering^82^ to replace the *E. coli* native *CadCBA* operon with a kanamycin resistance cassette. Chemically competent host cells of strain S1021^29^ were transformed with plasmid pKD119 as described above. Primers MM557 and MM559 with 5’ homology to regions of the genome flanking the *cadCBA* operon, were used to amplify the kanamycin resistance cassette from plasmid pKD13. The PCR product was gel-purified and transformed into 500 µL S1021 + pKD119 cells by electroporation and recovered overnight at 37 °C with shaking at 230 RPM in 4 mL SOC, then plated on 2xYT + 1.5% agar + kanamycin and incubated at 37 °C for 16 h.

Insertion of the kanamycin resistance cassette was verified by colony PCR using primers MM558 and MM560. Successful colonies were inoculated into 2xYT + kanamycin and grown up at 37 °C for 5 h before plating in parallel on 2xYT + 1.5% agar + kanamycin or tetracycline to verify successful curing of pKD119. Successful cultures were incubated in 2xYT + kanamycin for 2 h at 37 °C with the addition of 1 µL of F-plasmid donor culture, S1030^30^ or S2060^35^, and streaked on 2xYT + 1.5% agar + kanamycin, tetracycline and streptomycin. Since the loss of the *cadCBA* operon is associated with a slight fitness cost, Δ*cadCBA* cells were maintained with kanamycin throughout subsequent work to safeguard against contamination by strains lacking the Δ*cadCBA* deletion.

### Luciferase transcriptional activation assay

S536 and S1367 cells were transformed with APs and CPs as indicated in Supplementary table 2. Freshly saturated cultures of single colonies grown in Davis Rich Media^19^ (DRM) plus maintenance antibiotics were diluted 500-fold into DRM media with maintenance antibiotics in a 96-well deep-well plate (Axygen) and induced with indicated concentrations of arabinose (Gold Biotechnology) before incubation for 2 h at 37 °C with shaking at 230 RPM. 150 μL of cells per well were then transferred to a 96-well black-walled clear-bottomed plate with a transparent lid (Costar). 600 nm absorbance and luminescence were read at 15-min intervals over an 8-h kinetic cycle with shaking at 230 RPM between reads using a Tecan Spark multimode microplate reader (Tecan). Single read data were taken at peak luminescence value (4–5 h post-induction). OD_600_-normalized luminescence values were determined by dividing raw luminescence by background-subtracted (DRM only) 600 nm absorbance.

For phage-induced luciferase timecourse assay, S536 and S2060 cells were transformed with APs and diluted in DRM as described above. Cells were grown to an OD_600_ of 0.4 and were inoculated with selection phage at an initial titer of 5 × 10^4^ pfu/mL. 150 μL of cells per well were immediately transferred to a plate for luminescence and optical density reading in a kinetic cycle as described above.

### Phage propagation assay

S536 and S1367 cells were transformed with the AP(s) of interest as described above. Overnight cultures of single colonies grown in 2xYT media supplemented with maintenance antibiotics were diluted 1000-fold into DRM media with maintenance antibiotics and grown at 37 °C with shaking at 230 RPM to OD_600_ 0.4 exactly. Cells were infected with SP at an initial titer of 5 × 10^4^ pfu mL^−1^. Cells were incubated 16–18 h at 37 °C with shaking at 230 RPM, then centrifuged at 10,000 *g* for 2 min and the supernatant stored at 4 °C.

### Plaque assay

Saturated cultures of single colonies of strain S2208 grown in 2xYT media plus maintenance antibiotics were diluted 1000-fold into fresh 2xYT media with maintenance antibiotics and grown at 37 °C with shaking at 230 RPM to OD_600_ ~ 0.8 before use. SPs were serially diluted 100-fold (4 dilutions total) in H_2_O. 10 µL of phage dilution was added to 150 μL of cells and immediately mixed with 1 mL of liquid (55 °C) top agar (2xYT media + 0.6% agar) supplemented with 2% Bluo-gal (Gold Biotechnology). The mixture was then immediately pipetted onto one quadrant of a quartered Petri dish containing 2 mL of solidified bottom agar (2xYT media + 1.5% agar, no antibiotics) and allowed to solidify. Plates were incubated at 37 °C for 16–18 h. Titers were reported as one significant figure prior to calculating ratios.

### Phage-assisted continuous and non-continuous evolution

Cell preparation, PANCE, and PACE were carried out in DRM^61^. Chemically competent S536 or S1367 cells were transformed with AP(s) and DP6, plated on 2xYT media + 1.5% agar supplemented with 10 mM glucose (to suppress induction of mutagenesis from the P_BAD_ promoter) and maintenance antibiotics, and grown at 37 °C for 16 h. Colonies were picked into 500 µL DRM in a 96-well deep-well plate, and serially diluted 10-fold twelve times in DRM. Typically, eight colonies were selected. The plate was sealed with porous film and colonies allowed to grow at 37 °C with shaking at 230 RPM for 16–18 h.

For PACE, dilutions with OD_600_ ~ 0.4–0.8 were then used to inoculate an 80 mL DRM chemostat. The chemostat was continuously diluted with fresh DRM at a rate of ~1.5 chemostat volumes/h, maintaining a volume of 60-80 mL and an OD_600_ value between 0.8 and 1.0^17^.

Lagoons were continuously diluted from the chemostat culture at 1 lagoon volume/h and were induced with 10 mM arabinose ± 50 ng/mL aTc as indicated, for at least 2 h prior to infection with SP. For novel PACE campaigns, SPs were plaqued as described above and purified from single plaques by growing up ~8 h in fresh 2xYT media with maintenance antibiotics at 37 °C with shaking at 230 RPM. For continuations of previous PACE runs at increased stringency, 20 µL of lagoon samples from previous PACE endpoints were added to 2 mL of S2208 cells in mid-log growth phase and grown for ~4 h in 2xYT media plus maintenance antibiotics at 37 °C with shaking at 230 RPM. All selection phage cultures were centrifuged at 10,000*g* for 2 min and passed through a 0.22-μm PVDF Ultrafree centrifugal filter (Millipore) prior to use in PACE.

Lagoons were infected with purified SP at a starting titer of 10–10^6^ pfu/mL and maintained at a volume of 15 mL through the constant inflow of chemostat material and outflow of media waste at a rate of 0.5–3 lagoon volumes per hour. Arabinose and aTc concentrations within lagoons were maintained through constant inflow. 500-μL samples were taken at indicated times from lagoon waste lines. Samples were centrifuged at 10,000*g* for 2 min, and the supernatant was passed through a 0.22-μm PVDF Ultrafree centrifugal filter (Millipore) and stored at 4 °C.

Selection phage titers were determined by plaque assays using S2208 cells. Four or eight single plaques were PCR amplified as described above to characterize lagoon phage.

For PANCE, host strain dilutions with OD_600_ ~ 0.4–0.8 were further diluted to 50 mL in DRM plus appropriate antibiotics and grown up to OD_600_ ~ 0.4. 1 mL of cells were added to each well of a deep-well plate, allocating one well per replicate. Wells were induced with 10 mM arabinose if mutagenesis/drift plasmid was present and were inoculated with phage at 10^7^ pfu mL^−1^ unless otherwise indicated. Plates were grown up 16 h at 37 °C with shaking at 230 RPM. Plaques were amplified for characterization as described above. For restriction-enzyme-mediated phage characterization, 400 ng PCR-amplified phage DNA was cleaved with 0.4 µL HinfI (New England Biolabs) according to manufacturer’s instructions.

### Small-scale protein expression

BL21 DE3 cells (New England BioLabs) were transformed with expression plasmids (EPs) according to the manufacturer’s protocol. Single colonies were grown up overnight in 2xYT media plus maintenance antibiotics were diluted 1000-fold into fresh 2xYT media (2 mL) with maintenance antibiotics and grown at 37 °C with shaking at 230 RPM to OD_600_ 0.4. Cells were induced with 0.1 mM isopropyl-β-D-thiogalactoside (IPTG; Gold Biotechnology) or other indicated concentration and grown for a further 4 h at 37 °C with shaking at 230 RPM. 2 OD_600_ units of culture were isolated by centrifugation at 8000 *g* for 2 min. The resulting pellet was resuspended in 150 μL B-per reagent (Thermo Fisher Scientific) supplemented with protease inhibitor cocktail (Roche) and incubated at 25 °C for 15 min before centrifugation at 16,000 *g* for 2 min. The supernatant was collected as the soluble fraction. The pellet was resuspended in an additional 150 μL B-per reagent to obtain the insoluble fraction. To 37.5 μL of each fraction was added 12.5 μL 4x NuPage LDS sample buffer (Thermo Fisher Scientific). Fractions were vortexed and incubated at 95 °C for 10 min. 12 μL (soluble fraction) or 5 μL (insoluble fraction) was loaded per well of a Bolt 4–12% Bis-Tris Plus (Thermo Fisher Scientific) pre-cast gel. 5 μL of Precision Plus Protein Dual Color Standard (Bio-Rad) was used as a reference. Samples were separated by electrophoresis at 200 V for 30 min in Bolt MES SDS running buffer (Thermo Fisher Scientific). Gels were stained with InstantBlue reagent (Expedeon) for –16 h and destained for 1 h in water before imaging with a G:Box Chemi XRQ (Syngene).

### Periplasmic extraction

Periplasmic extraction was carried out as previously described^83^. Briefly, 100 mL of cells at OD_600_ = ~1 were pelleted by centrifugation at 3000 *g*, drained, and carefully resuspended in 1 mL TSE buffer (200 mM Tris-HCl pH 8.0, 500 mM sucrose, 1 mM EDTA) plus protease inhibitor cocktail (Roche). Cell pellets were incubated on ice for 30 min and supernatant (periplasm extract) was separated from cell pellet (spheroplast) by centrifugation at 16,000 *g* for 30 min at 4 C. Samples were analyzed by SDS-PAGE and Western blot.

### Western blot analysis

Following SDS-PAGE, proteins were transferred to a PVDF membrane using an iBlot 2 Gel Transfer Device (Thermo Fisher Scientific) according to the manufacturer’s protocol. The membrane was blocked in SuperBlock Blocking Buffer (Thermo Fisher Scientific) for 1 h at room temperature, then incubated overnight at 4 °C in SuperBlock Blocking Buffer (Thermo Fisher Scientific) plus one or more of the following, as indicated: mouse anti-6xHis (abcam ab18184; 1:2000 dilution), mouse anti-c-ABL (Sigma-Aldrich A5844; 1:2000 dilution), mouse anti-MBP (abcam ab65, 1:5000 dilution) and rabbit anti-GroEL (Sigma-Aldrich G6532; 1:20,000 dilution). If both primary and loading control antibodies were mouse-derived, as in Supplementary Fig. 6g, membrane was cut according to expected MW of target and membrane halves were incubated separately in primary antibodies, as indicated. The membrane was washed 3x with TBST (TBS + 0.5% Tween-20) for 10 min each at room temperature, then incubated with IRDye-labeled secondary antibodies goat anti-mouse 680RD (LI-COR 926–68070) and donkey anti-rabbit 800CW (LI-COR 926–32213) diluted 1:5000 for 1 h at 25 °C. The membrane was washed 3x with TBS as before. Imaging was performed using the Odyssey Imaging System (LI-COR).

Band densities were quantified using ImageJ and normalized to reference bands to control for loading. Uncropped blot images can be found in Supplementary Figs. 9, 15 and 16.

### Large-scale protein expression and purification

BL21 DE3 cells transformed with EPs of interest were grown in LB or 2xYT media containing maintenance antibiotics overnight from single colonies. Cultures were diluted 1000-fold into fresh 2xYT media (1 L) with appropriate antibiotics and grown up at 37 °C with shaking at 230 RPM to OD_600_ ~ 0.4–0.5. Cells were induced with 50 uM IPTG and grown for a further 16–18 h at 16 °C with shaking at 200 RPM. Cells were isolated by centrifugation at 8000*g* for 10 min and washed 1x with 20 mL TBS (20 mM Tris-Cl, 500 mM NaCl, pH 7.5). The resulting pellet was resuspended in 12 mL B-per reagent supplemented with EDTA-free protease inhibitor cocktail (Roche) and incubated on ice for 30 min with regular vortexing, before centrifugation at 16,000 *g* for 18 min. The supernatant was decanted into a 50 mL conical tube and incubated with 1 mL of TALON Cobalt (Clontech) resin at 4 °C with constant agitation for 2 h, after which the resin was isolated by centrifugation at 500 *g* for 5 min. The supernatant was decanted, and the resin resuspended in 4 mL binding buffer (50 mM NaH_2_PO_4_, 300 mM NaCl, 20 mM imidazole, pH 7.8) and transferred to a column. The resin was washed 4x with 4 mL binding buffer before protein was eluted with 2 ×1 mL of binding buffer containing increasing concentrations of imidazole (50–300 mM in 50 mM increments). The fractions were analyzed by SDS-PAGE. Combined pure fractions were buffer-exchanged with TBS and concentrated using an Amicon Ultra-15 centrifugal filter unit (10,000 molecular weight cutoff; Millipore), then stored at 4 °C for up to one week or else snap-frozen in liquid nitrogen for –80 °C storage. Total protein was quantified using a BCA protein assay kit (Pierce) using BSA standards (Bio-Rad). Quantification of specific bands, where necessary, was carried out by gel densitometry using ImageJ software with comparison to reference lanes loaded with known quantities of BSA (Bio-Rad).

### ELISA

Pre-blocked high-capacity streptavidin-coated 96-well clear plates (Pierce) were washed 3X with 200ul/well TBST and incubated overnight at 4C with purified biotin-tagged protein (Her2, TGFB1, AcroBiosystems; H98 peptide, biotin–GGGGSLLGPYELWELSH, GenScript Custom Peptide) diluted as indicated in TBS. After overnight incubation, wells were washed 3X with 200ul/well TBST and incubated at room temperature for 2 h with 25ug/mL purified antibody fragments in TBS, 50 µL per well. Wells were washed 3X with 200ul/well TBST and incubated for a further 45 min with protein a-HRP (Thermo Fisher Scientific 101023, 1:2000 dilution) in TBS. Finally, wells were washed 4X with 200ul/well TBST, then developed with 50 µL/well 1-Step Ultra TMB-ELISA Substrate (Thermo Fisher Scientific) for 90 seconds. Quenching was carried out with 50 µL/well 2 M H_2_SO_4_ and 450 nm absorbance was read using a Tecan Spark multimode microplate reader. Values were normalized by subtracting the mean value at 0 nM antigen for each variant and dividing all values by the maximum mean value of the unmutated TR control. EC_50_ values were calculated using a sigmoidal 4-point linear regression in Prism 8.

### Microscale thermophoresis

MST was carried out using the Monolith NT.115 system (Nanotemper) according to the manufacturer’s instructions. H98 peptide (GenScript) was resuspended in DMSO and diluted in TBS-T to a final concentration of 6.25% DMSO. Trastuzumab and variant scFvs were diluted in TBS-T to a final concentration of 5 nM and fluorophore-tagged with cy3-conjugated anti-6XH antibody (Rockland Antibodies & Assays) at a 1:1 molar ratio. Reads were carried out using Monolith.NT automated capillary chips (Nanotemper). Data were analyzed with built-in MO.Control and MO.Affinity Analysis software, version 2.3.

### Growth timecourse assay

For phage-based growth timecourse assays, S1367 cells were transformed with permissive accessory plasmid pJC175e^19^. Freshly saturated cultures of single colonies grown in DRM^19^ media plus maintenance antibiotics were diluted 1000-fold into DRM media with maintenance antibiotics until OD_600_ ~ 0.1 was reached. Biological replicates were infected with phage at indicated initial titers, and 150 μL of cells per well were immediately transferred to a 96-well black-walled clear-bottomed plate with a transparent lid (Costar). 600 nm absorbance and luminescence were read at 10-min intervals over a 9-h kinetic cycle with shaking at 230 RPM between reads using a Tecan Spark multimode microplate reader (Tecan).

For plasmid-based growth timecourse assays, S1367 cells were transformed with pJC175e and CPs as indicated in Supplementary table 2. Freshly saturated cultures of single colonies grown in DRM^19^ media plus maintenance antibiotics were diluted 500-fold into DRM media with maintenance antibiotics in a 96-well deep-well plate (Axygen) and induced with indicated concentrations of arabinose (Gold Biotechnology) before incubation for 2 h at 37 °C with shaking at 230 RPM. 150 μL of cells per well were then transferred to a 96-well black-walled clear-bottomed plate with a transparent lid (Costar). 600 nm absorbance and luminescence were read at 10-min intervals over a 9-h kinetic cycle with shaking at 230 RPM between reads using a Tecan Spark multimode microplate reader (Tecan).

### Protein melt temperature assay

Melt temperatures were determined using the Protein Thermal Shift Dye Kit (Life Technologies) according to the manufacturer’s protocols. A CFX96 Real-Time PCR Detection System (Bio-Rad) was used to monitor fluorescence.

### Reporting summary

Further information on research design is available in the Nature Research Reporting Summary linked to this article.

## Supplementary information

Supplementary Information

Reporting Summary

## Data Availability

Materials such as plasmids and maps will be made available upon reasonable request. Cited crystal structure data are available in the Protein Database under entry IDS 1J85 (https://www.rcsb.org/structure/1J85) and 1N8Z (https://www.rcsb.org/structure/1N8Z). Source data are provided with this paper. No code was used in this manuscript. No large datasets were generated in this manuscript. Source data are provided with this paper.

## Source data

Source Data
