# Author Correction: Disulfide-compatible phage-assisted continuous evolution in the periplasmic space

**DOI:** 10.1038/s41467-021-27102-0

**Published:** 2021-11-17

**Authors:** Mary S. Morrison, Tina Wang, Aditya Raguram, Colin Hemez, David R. Liu

**Affiliations:** 1 Merkin Institute of Transformative Technologies in Health Care, Broad Institute of Harvard and MIT, Cambridge, MA 02142 USA; 2 Department of Chemistry and Chemical Biology, Harvard University, Cambridge, MA 02138 USA; 3 Howard Hughes Medical Institute, Harvard University, Cambridge, MA 02138 USA

**Keywords:** Genetic engineering, Protein design, Synthetic biology, Chemical biology

Correction to: *Nature Communications* 10.1038/s41467-021-26279-8, published online 13 October 2021.

In this article the grant number R01 EB027793 relating to NIH was omitted. The original article has been corrected.

