# Supplementary material for: Disulfide-compatible phage-assisted continuous evolution in the periplasmic space
Source: Nat Commun. 2021 Oct 13;12:5959. doi: 10.1038/s41467-021-26279-8 (PMC8514426; doi:10.1038/s41467-021-26279-8)
Supplement: Supplementary file 1 — Supplementary Information [file 41467_2021_26279_MOESM1_ESM.pdf]

Supplementary Information

Disulfide-compatible phage-assisted continuous evolution in the periplasmic space

Mary S. Morrison[1,2,3], Tina Wang[1,2,3], Aditya Raguram[1,2,3], Colin Hemez[1,2,3] & David R. Liu[1,2,3*]

[1] Broad Institute of Harvard and MIT, Cambridge, MA, 02142

[2] Department of Chemistry and Chemical Biology, Harvard University, Cambridge, MA, 02138

[3] Howard Hughes Medical Institute, Harvard University, Cambridge, MA, 02138

* Correspondence should be addressed to David R. Liu: drliu@fas.harvard.edu

Supplementary Figure 1	Optimization of P_{cadBA}	3
Supplementary Figure 2	Effect of host *cadCBA* operon deletion	4
Supplementary Figure 3	PANCE of YibK	5
Supplementary Figure 4	Western blots show YibK-SH2 periplasmic localization and disulfide-mediated covalent bond formation	6
Supplementary Figure 5	Characterization of the initial pPACE system	8
Supplementary Figure 6	Split-intein signal sequence allows regulation of antibody export to the periplasm	9
Supplementary Figure 7	Design of periplasmic PACE of trastuzumab scFv	11
Supplementary Figure 8	Characterization of second-generation pPACE system	12
Supplementary Figure 9	Second-generation periplasmic PACE of the Ω-graft antibody	14
Supplementary Figure 10	Periplasmic PACE of trastuzumab	16

Supplementary Figure 11	Periplasmic PACE of trastuzumab scFv at high stringency produces no novel converged mutations	17
Supplementary Figure 12	Trastuzumab and evolved variants require disulfides for activity	18
Supplementary Figure 13	Phage-based and plasmid-based periplasmic scFv expression does not impair host cell growth rate	19
Supplementary Figure 14	Microscale thermophoresis analysis of trastuzumab scFv (TR) and variants 1.1 and 3.2	20
Supplementary Figure 15	Soluble expression and thermostability characteristics of evolved trastuzumab variants 1.1 and 3.2	21
Supplementary Figure 16	Trastuzumab scFv and evolved variants used in biochemical characterizations	23
Supplementary Table 1	Plasmid names, strains, phage and arabinose induction concentrations used in this work	24
Supplementary Table 2	Plasmids used in this work	26
Supplementary Table 3	Selection phage used in this work	28
Supplementary Table 4	Primers used in this workwork	29
Supplementary Table 5	Properties of trastuzumab scFv and evolved variants determined by MST analysis	29
Supplementary Discussion	Background bands	30
Supplementary Note 1	DNA sequences used in this work	31
Supplementary References		37

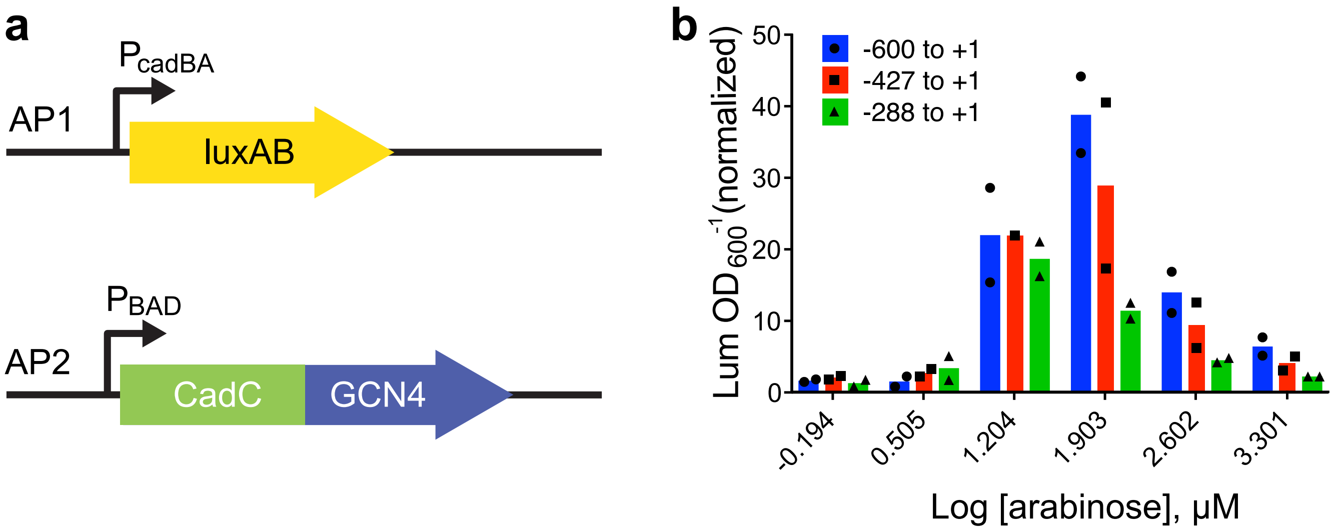

Supplementary Figure 1. Optimization of P_{CadBA}. (a) Overview of CadC luciferase-based transcriptional activation reporter. (b) P_{CadBA} optimization. AP1 constructs incorporate three different spans of upstream untranslated regions of the promoter P_{CadBA}, which is activated by CadC dimerization. CadC molecules bind Cad1 and Cad2 DNA motifs at positions –144 to –112 bp and –89 to –59 bp respectively, but retention of 5' UTR up to base –600 leads to maximal signal-to-noise ratio across multiple levels of arabinose-mediated P_{BAD} induction of CadC linked to the dimeric leucine zipper GCN4. Y-axis shows the ratio of OD_{600}-normalized luminescence induced by wild-type GCN4 leucine zipper to OD_{600}-normalized luminescence induced by GCN4 monomeric variant 7P14P, which does not dimerize and is not expected to drive P_{CadBA} activation. Bar valuesrepresent mean of two biological replicates. Source data are provided as a Source Data file.

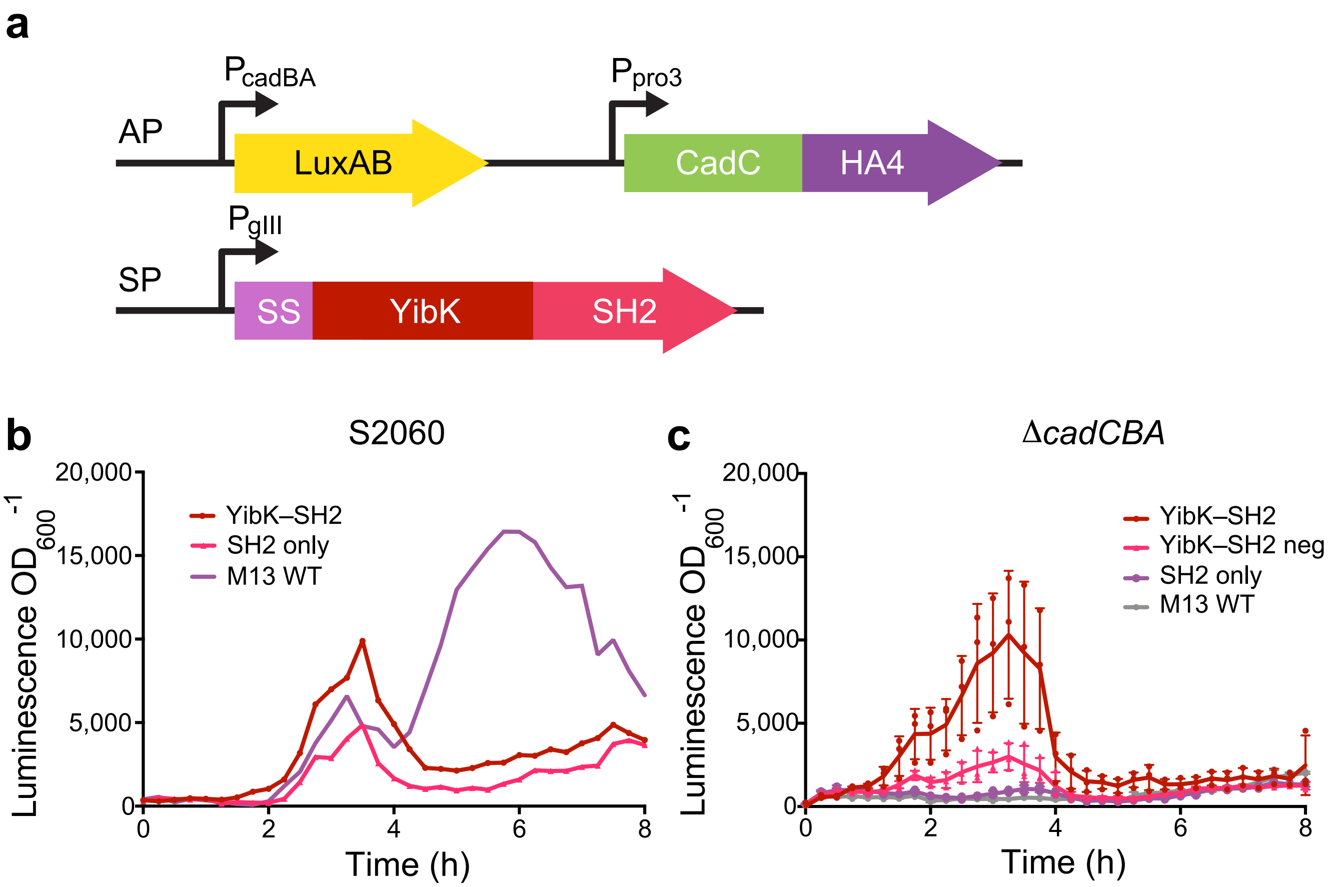

Supplementary Figure 2. Effect of host *cadCBA* operon deletion. (a) Overview of CadC luciferase-based transcriptional activation reporter of YibK dimerization. The monobody HA4 binds and recruits SH2 with high affinity; see Fig. 2a. (b) Phage-induced luciferase transcriptional activation time course in unmodified host strain S2060 shows background signaling mediated by wild-type M13 phage infection (no YibK expression). A single replicate is shown. This assay was repeated once with similar results. (c) Phage-induced luciferase transcriptional activation time course in a PACE host strain with deletion of the native *cadCBA* operon shows no M13-mediated background P_{cadBAc} signaling. 'Neg' indicates monomeric YibK mutant V139R. Data reflect mean and s.d. of three biological replicates. Individual data points are also shown. Source data are provided as a Source Data file.

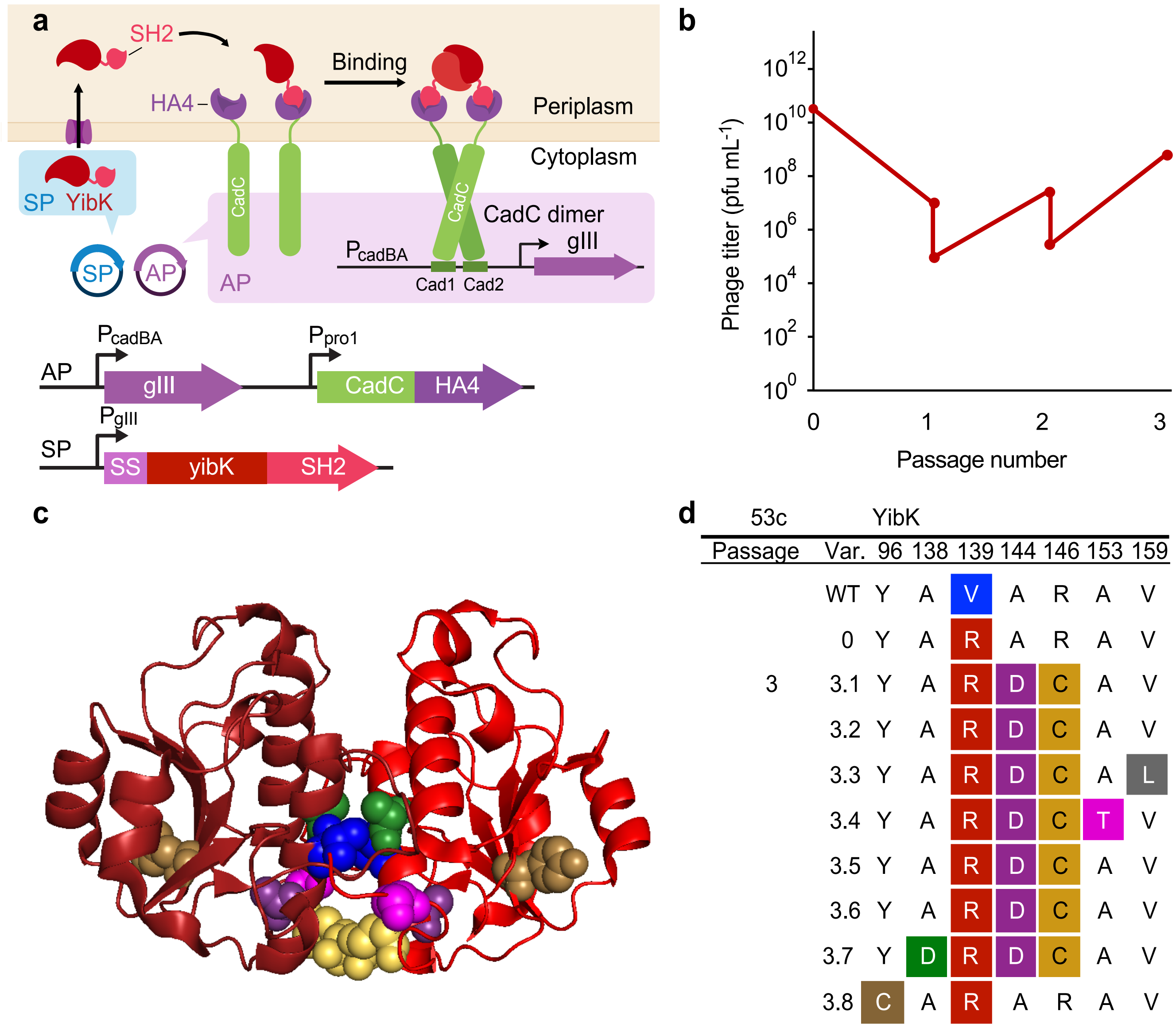

Supplementary Figure 3. PANCE of YibK. (a) Periplasmic PACE circuit to correct monomeric binding mutant in YibK. The SH2-binding HA4 monobody is used to recruit the SH2-fused YibK species to CadC. (b) Phage titers through 24-hour cycles of PANCE. (c) Positions mutated in YibK PANCE are shown in the YibK dimer crystal structure (PDB ID: 1J85)[1]. Positions are colored to correspond to YibK variant sequences shown in (d). Position R146 (yellow) is in close proximity to R146' on the opposing subunit, while positions A138 and V139 make mutual contacts (A138:V139', A138':V139) at the dimer interface. Position V159 falls in an unstructured region not captured by the crystal structure (PDB ID: 1J85)[1].[1]. Source data are provided as a Source Data file..

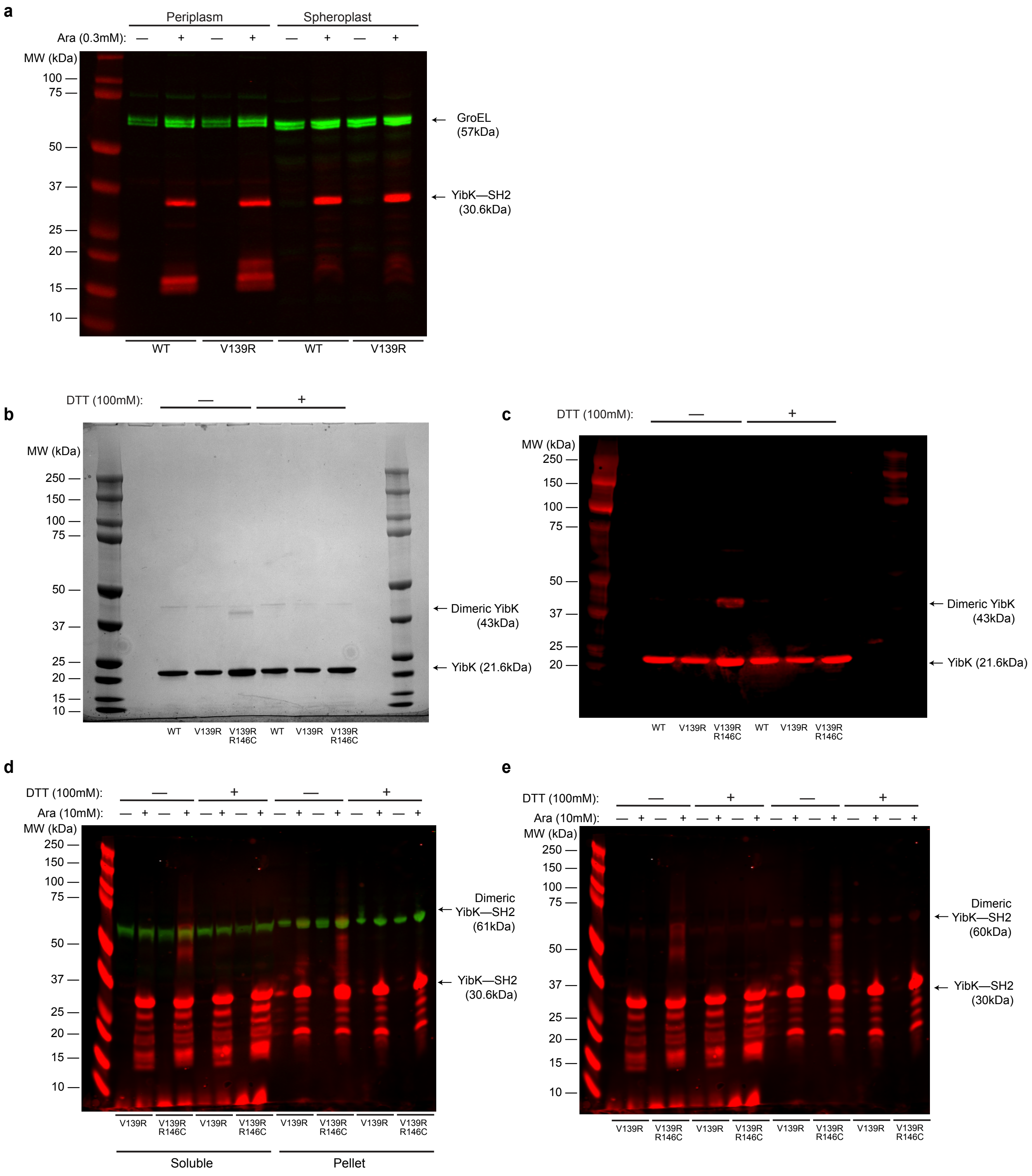

Supplementary Figure 4. Western blots show YibK-SH2 periplasmic localization and disulfide-mediated covalent bond formation. (a) Periplasmic extraction following arabinose (abbreviated Ara) induction of YibK–SH2 expression from P_{BAD}. Red channel: anti-c–ABL, which recognizes the SH2 domain. Green channel: GroEL reference (57kDa). This experiment was repeated once with similar results. (b) Coomassie of IMAC-purified 6XHis-tagged YibK (21.6kDa). The covalent dimeric species (43kDa) is visible for the V139R R146C

variant and is destroyed by addition of a reducing agent, dithiothreitol (DTT). This assay was repeated once, with similar results. (c) Full western blot shown in Fig. 2e, showing purified YibK protein as in (b). The 43kDa band representing the dimeric species is visible in the red channel. Red channel: anti-6XHis. Green channel: GroEL reference (57kDa). (d) Western blot of whole-cell lysate showing a 60-kDa band expected to represent a covalent YibK–SH2 dimer that is dependent on mutation R146C and that is destroyed by addition of DTT. The monomeric YibK–SH2 construct is 30 kDa. Red channel: anti-c–ABL, which recognizes the SH2 domain. Green channel: GroEL reference (57kDa). (e) Western blot shown in (d) with green GroEL (57kDa) reference channel hidden, to better reveal 60kDa band. This experiment was repeated once with similar results. Background or non-target bands in this figure are discussed in Supplementary Discussion. Source data are provided as a Source Data file.

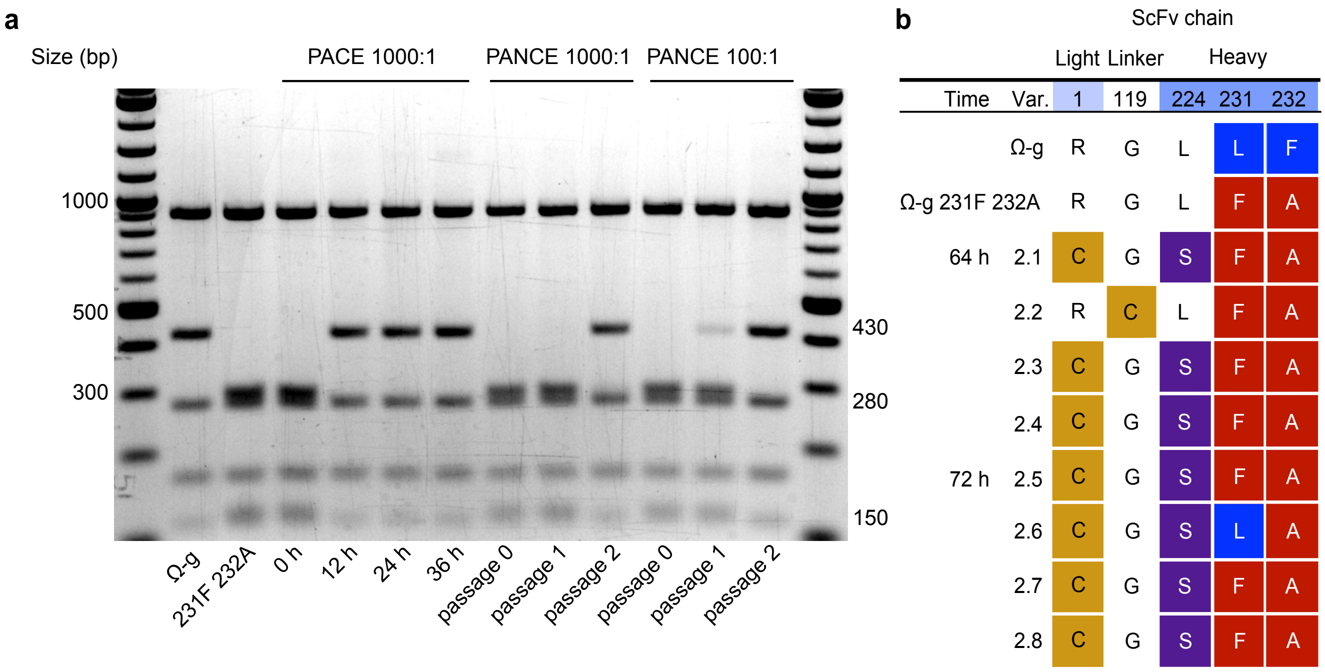

Supplementary Figure 5. Characterization of the initial pPACE system. Ω-graft (Ω-g)–SH2 phage evolution in original selection architecture. (a) Restriction-enzyme-mediated characterization of monoclonal phage (lanes 2–3) and PANCE and PACE outputs. In these selections, no mutagenesis was induced, and phage populations were seeded with binding mutant L231F F232A and unmodified Ω-g in the indicated ratios. PANCE was passaged by 1:100 dilution of phage. HinfI (5’–G$^{\vee}$ANTC) cleaves the gene encoding the L231F F232A Ω-graft mutant (5’–GG$^{\vee}$ATTCGCT), resulting in cleavage of 430-bp band into 280-bp and 150-bp bands, but does not cleave the unmodified Ω-graft sequence (5’–GGACTTTTT). Residual bands at ~860bp, ~270bp and ~186bp are believed to be the result of cryptic cleavage sites in the original 1896-bp amplicon. This assay was repeated once with similar results. (b) Phage Ω-graft sequences resulting from PACE with mutagenesis showing mutations to cysteine at the N-terminus (position R1 following the cleaved signal sequence) and linker (position G119), and poor enrichment of the F231L reversion. Var. = variant. Linker and N-terminal cysteine residues are highlighted in gold. Source data are provided as a Source Data file.

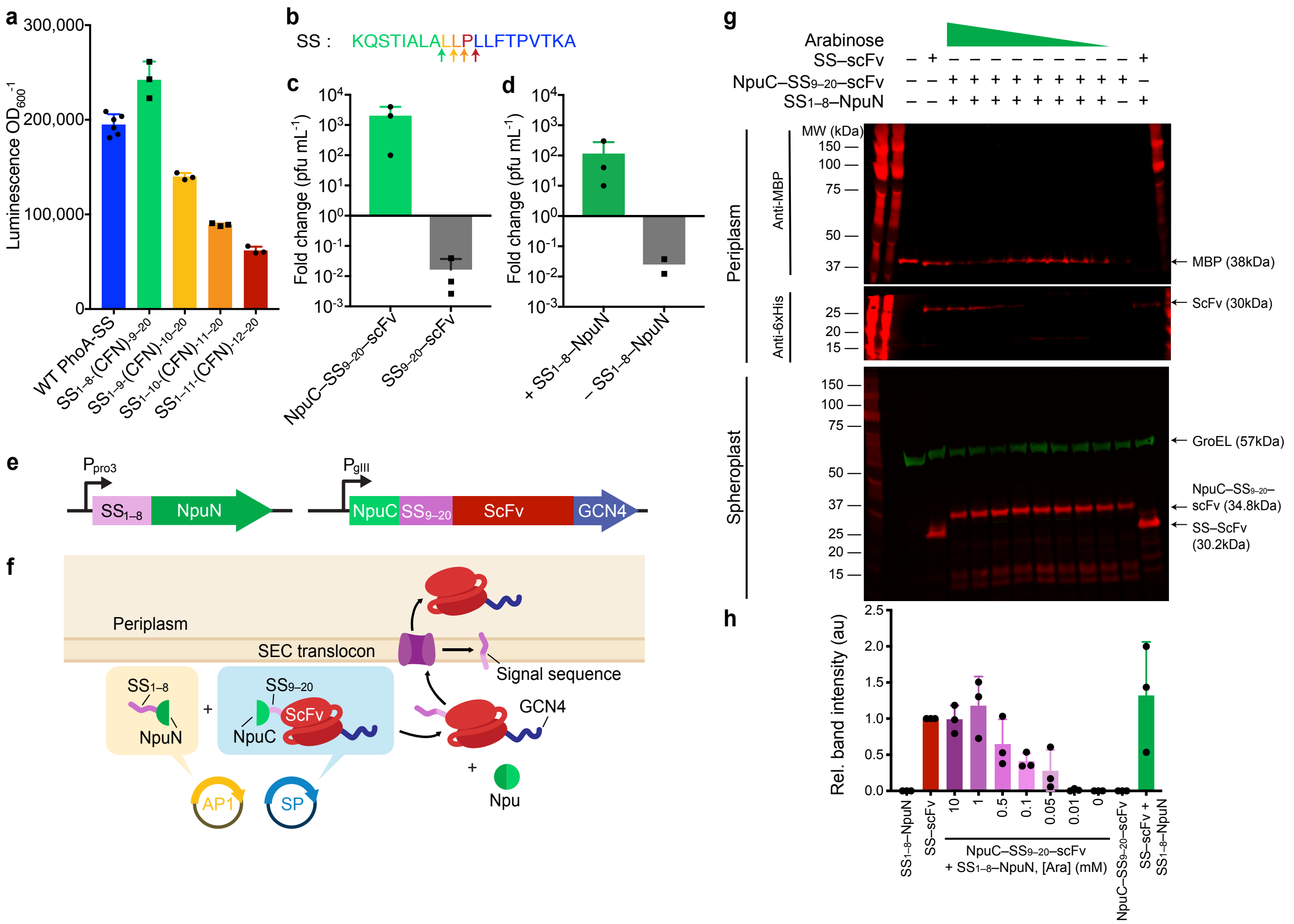

Supplementary Figure 6. Split-intein signal sequence allows regulation of antibody export to the periplasm. (a) Transcriptional activation assay to evaluate disruption of P_{CadBA} signaling caused by insertion into the signal sequence of the CFN scar that is the product of intein-mediated cleavage into the signal sequence. Residues CFN can be inserted into the SS without loss of periplasmic scFv-mediated transcriptional activation of $P_{CadBA.}$ Bar values reflect mean and s.d. of three biological replicates. (b) Signal sequence (SS), showing locations of intein insertion. (c-d) Selection phage enrichment assays. Intein NpuC domain is required for reconstitution of the SS and P_{CadBA}–gIII transcription activation and phage propagation (c). Likewise, phage do not propagate when fragment $SS_{1–8}$–NpuN is not supplied (d). Bar values and error bars reflect mean and s.d. of three (c, d treatment + $SS_{1–8}$–NpuN) or two (d treatment) biological replicates, carried out on separate days. (e-f) Overview of intein-mediated split SS system. SS residues 1–8 and NpuN-terminal domain are provided constitutively by an accessory plasmid (AP). (g-h) Western blot of periplasmic extraction from BL21*DE3 cells, showing intein-mediated periplasmic scFv expression. Expression of $SS_{1–8}$–NpuN was driven by arabinose-inducible promoter P_{BAD} and induced with multiple concentrations of arabinose, with a constant level of IPTG (0.1mM) inducing expression of NpuC–$SS_{9–20}$–scFv. ScFv with full-length SS was used as a positive control. Single transformants, lacking plasmids encoding either $SS_{1–8}$–NpuN or NpuC–$SS_{9–20}$–scFv, were used as negative controls. Concentrations are shown in (h) corresponding with lanes in (g). Maltose-binding protein (MBP) was used as a periplasmic fraction loading control. Results of three replicates carried out on separate days were quantified by densitometry and were

normalized first to the loading control, then to the value of positive control SS–scFv. Bar values and error bars reflect mean and s.d. of three biological replicates carried out on separate days. Background or non-target bands in this figure are discussed in Supplementary Discussion. Source data are provided as a Source Data file.

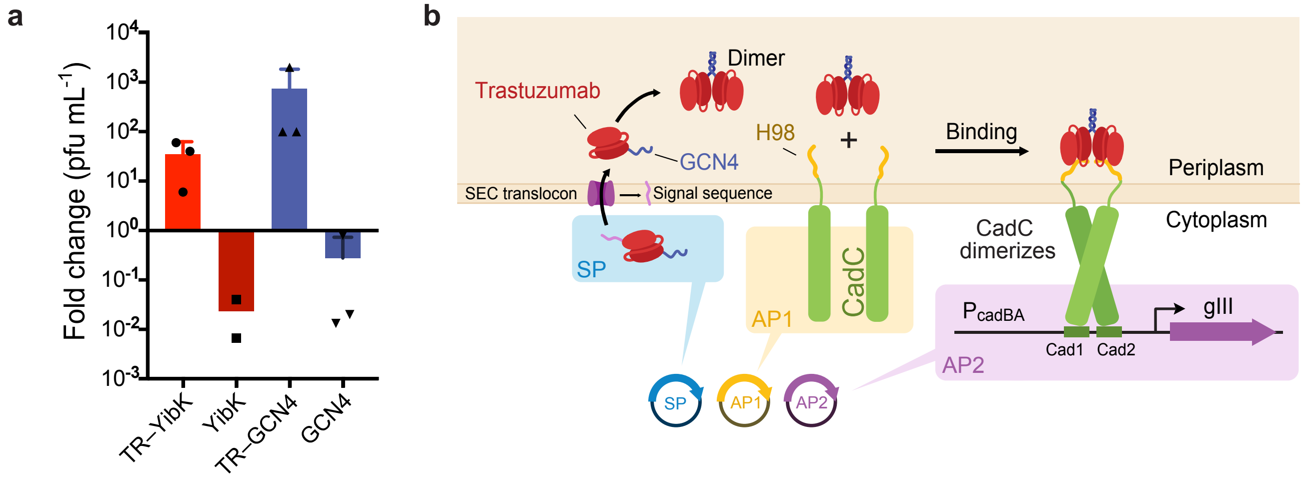

Supplementary Figure 7. Design of periplasmic PACE of trastuzumab scFv. (a) Phage enrichment assay evaluating domains to direct the dimerization of trastuzumab (TR). Dimerization with YibK imposes a fitness cost to phage when compared to dimerization with GCN4, likely due to its larger size. Bar values and error bars represent mean and s.d. of two (YibK treatment) or three (all other treatments) biological replicates conducted on separate days. Source data are provided as a Source Data file. (b) Overview of trastuzumab periplasmic selection.

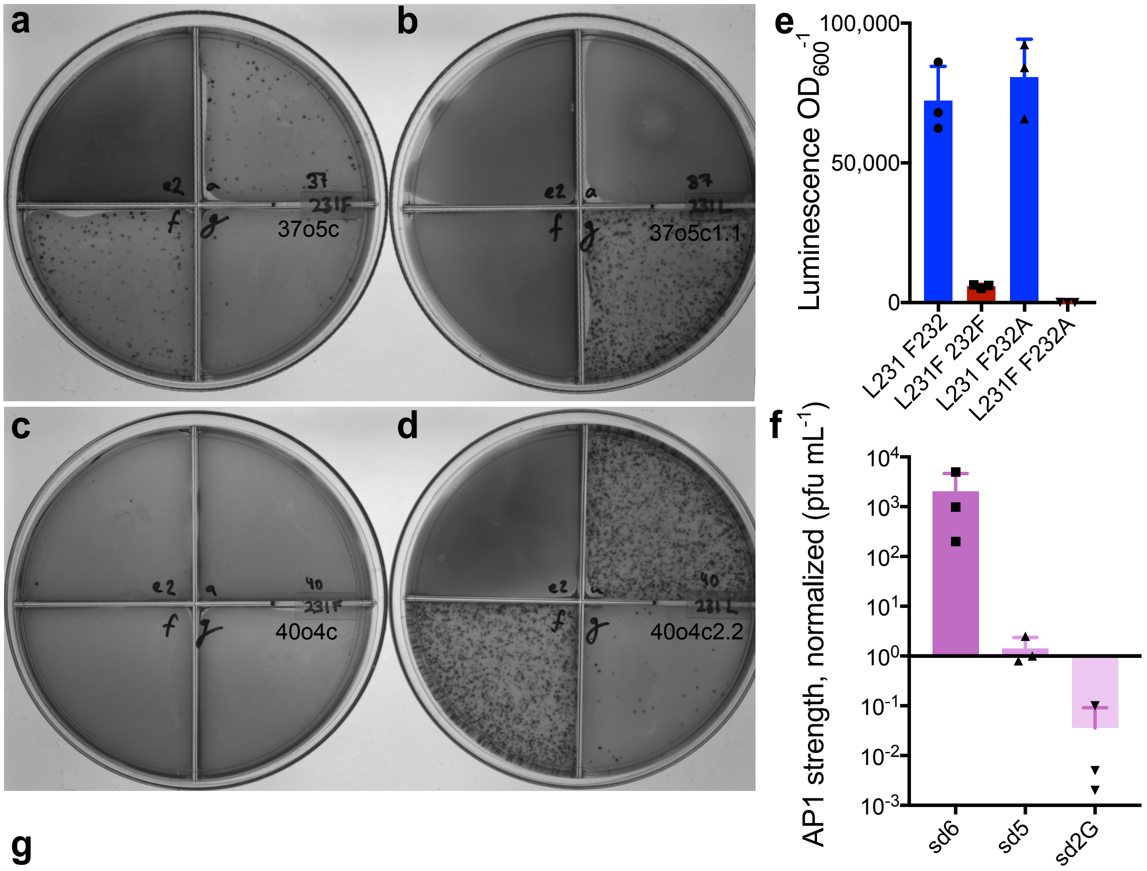

g

Plate	Phage	Sequence	AP1 construct	Plaque strength
a	37o5c	R1C L231F F232A	pMM116a	++
			pMM116e2	+++++
			pMM116f	++
			pMM116g	+
b	37o5c1.1	R1C F232A	pMM116a	+++++
			pMM116e2	+++++
			pMM116f	+++++
			pMM116g	+++
c	40o4c	R1C L231F F232A	pMM116a	-
			pMM116e2	+
			pMM116f	-
			pMM116g	-
d	40o4c2.2	R1C F232A	pMM116a	+++
			pMM116e2	+++++
			pMM116f	+++
			pMM116g	++

Supplementary Figure 8. Characterization of second-generation pPACE system. (a–d) Phage enrichment assays showing stringency parameters of various AP1 constructs. Each quadrant represents 10 µL undiluted SP enriched overnight on the indicated AP1. AP1 constructs differ by strength of ribosome-binding site (RBS) directing gene III transcription from P_{CadBA}. All phage contain the pre-encoded R1C mutation to direct covalent dimerization. Phage with the 37o5c construct design have full-length SS, while those with the 40o4c construct have the split-intein SS. Phage are visible as dark spots. (e) Luciferase-based transcriptional activation assay shows that L231F is responsible for loss of binding in the L231F F232A mutant. Bar values and error bars reflect the mean and s.d. of three biological replicates. (f) Relative strengths of AP1 constructs as measured by relative enrichment of phage 37o5c variant 1.1 (L231). Enrichment values are normalized to AP1 construct pMM116a1, which encodes an

sd8 RBS and represents an enrichment score of 1. Bar values and error bars represent mean and s.d. of three biological replicates carried out on separate days. (g) Table summarizing the results shown in (a–d) including phage construct genotypes. Source data are provided as a Source Data file.

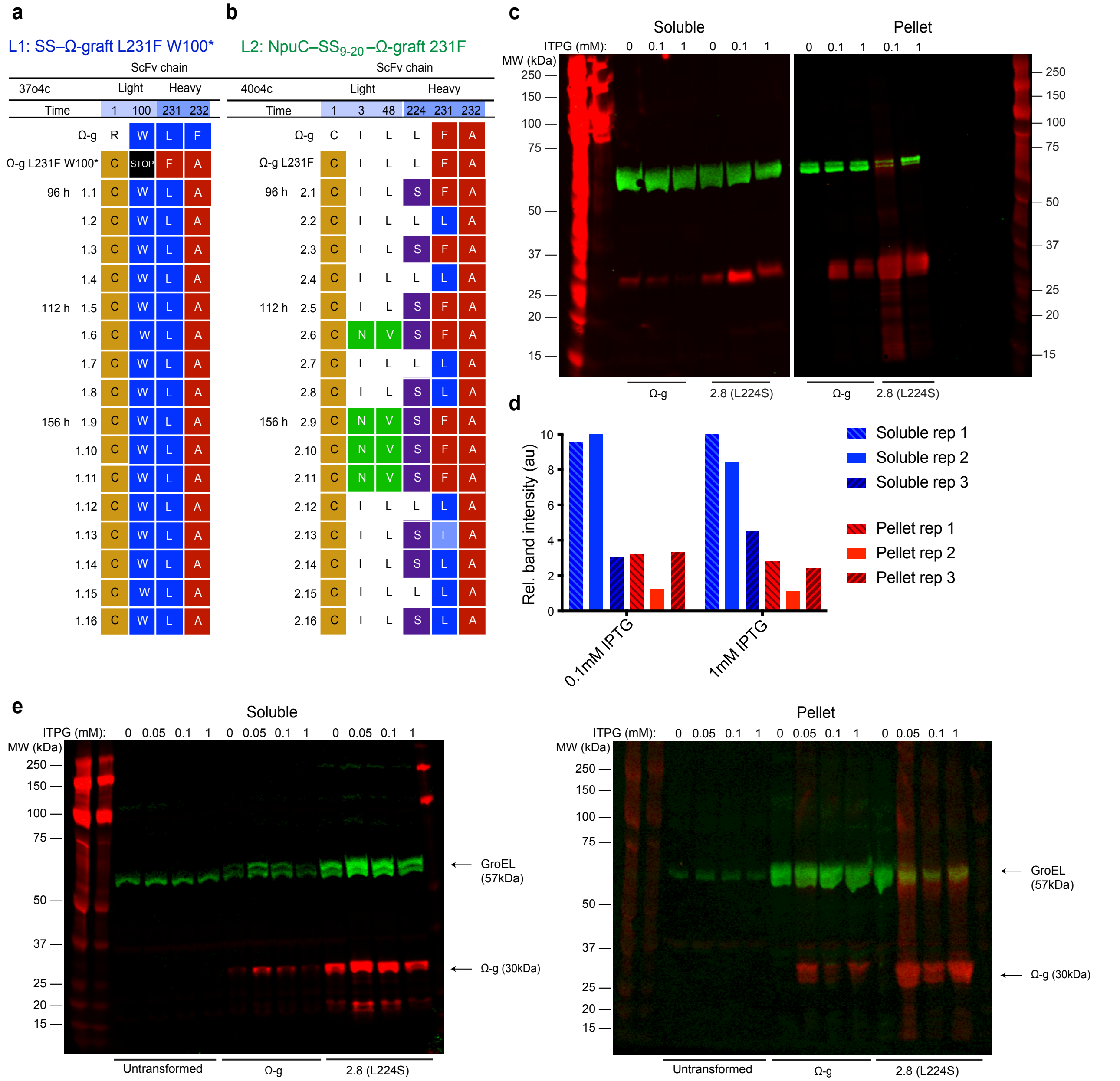

Supplementary Figure 9. Second-generation periplasmic PACE of the Ω-graft antibody. (a–b) Ω-graft (Ω-g) selection phage sequences show convergent evolution of mutations during PACE. Use of full-length SS (37o5c) appears to select solely for correction of the stop codon and L231F binding mutant, while use of a split intein SS (40o4c) selects for correction of both the binding mutant and L224S. The roles of I3N and L48V were not characterized. A single replicate of each population also enriched 100W and 231L (replicate of (a)) or 224S and 231L (replicate of (b)). (c) Full Western blot from main text Fig. 4i showing the effect of mutation L224S on soluble and insoluble expression levels across multiple IPTG concentrations when scFvs are expressed from P_{T7Lac} in BL21*DE3 cells. Red channel: anti-6XH. Green channel: anti-GroEL. (d) Gel densitometry quantification of bands in (c) and in an additional biological replicate experiment carried out on a separate day, normalized to GroEL reference. The value for the variant 2.8 (L224S) band was then normalized again to the value for Ω-g. (e)

Additional western blot data showing expression of Ω-g and variant 2.8 from the IPTG-inducible promoter P_{T7Lac} in BL21*DE3 cells, at multiple levels of induction with IPTG, including untransformed as well as uninduced controls. Background bands in this figure are discussed in Supplementary Discussion. This experiment was repeated a total of three times. Source data are provided as a Source Data file.

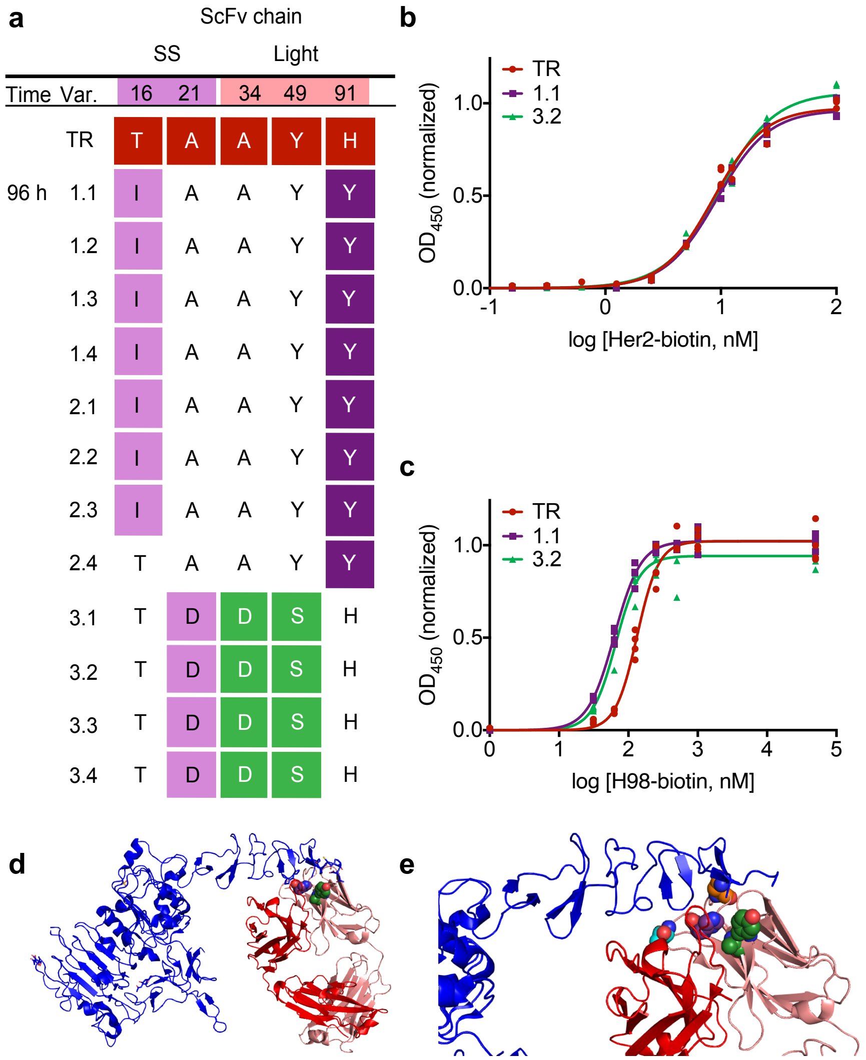

Supplementary Figure 10. Periplasmic PACE of trastuzumab. (a) Individual phage variants (Var.) emerging from PACE at 96 h show strong convergence of two distinct genotypes. The signal sequence (SS) directs periplasmic export of the scFv. Red, unmutated residue in TR, lilac, mutations affecting the SS; purple; mutations characteristic of variant 1.1; green; mutations characteristic of variant 3.2 (b) ELISA shows no significant change in affinity of trastuzumab variants 1.1 and 3.2 for Her2 compared to trastuzumab (TR). Data reflect mean and s.d. of three technical replicates. This assay was repeated once with a separate protein preparation and yielded similar results. (c) Full ELISA against mimetic peptide H98 shown in main text Fig. 5f, showing data points at far ends of the H98 dilution series. Four technical replicates are shown. This experiment was repeated three times with similar results. Mean IC_{50} values and s.d. from all four experiments are provided in main text Table 1. (d) Crystal structure of trastuzumab fragment bound to Her2, showing the location of PACE-evolved mutations[2]. Mutations are shown as spheres and are colored as in (a). (e) Close-up of (d), also showing residues N30 (orange) and T94 (cyan). These residues are predicted to be directly involved in binding of the trastuzumab light chain to the Her2 mimetic peptide H98[3]. Crystal structures adapted from PDB ID: 1N8Z[2].. All ELISA curves were fitted using a sigmoidal four-point log nonlinear fit (Prism 8). Source data are provided as a Source Data file.

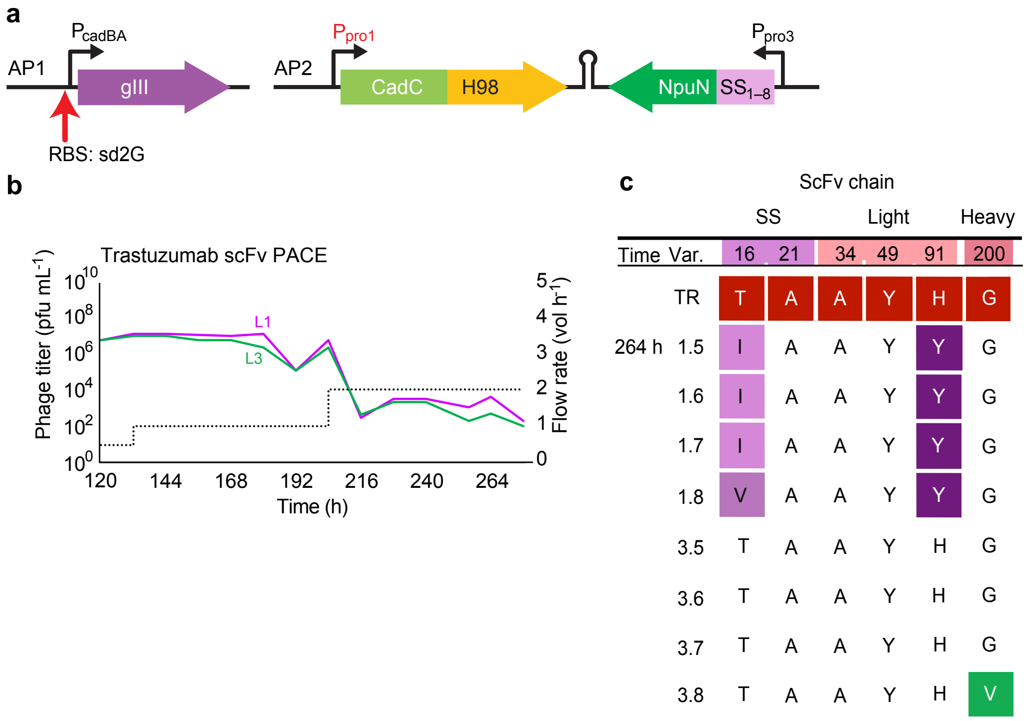

Supplementary Figure 11. Periplasmic PACE of trastuzumab scFv at high stringency produces no novel converged mutations. (a) Periplasmic PACE selection with increased stringency, seeded from lagoons 1 and 3 of trastuzumab scFv at 120 hours. On AP1, ribosome binding site (RBS; red arrow) strength driving pIII translation has been reduced from sd2 (0.001 relative expression units compared to SD8) to sd2G (0.0004 relative expression units compared to SD8)[4]. This change is expected to increase overall selection pressure. On AP2, constitutive promoter P_{pro3} (0.017 relative promoter units compared to promoter P_{ProD}) has been replaced with constitutive promoter P_{pro1} (0.009 relative promoter units compared to promoter P_{ProD}; highlighted in red)[5]. This change is expected to reduce antigen availability of CadC–H98 and increase selective pressure for high affinity to H98. (b) Trastuzumab scFv pPACE carrying populations L1 and L3 (main text Fig. 5, Supplementary Fig. 1) forward from 120 h timepoint into the more stringent selection conditions shown in (a). Drift was applied from 120 h to 168 h, resulting in a period of low selective pressure to increase the size of the scFv library available for selection. (c) Individual phage emerging from high-stringency pPACE of trastuzumab scFv at 256 h show no converged mutations not present in hours 1-120 of pPACE experiment (main text Fig. 5, Supplementary Fig. 1). Each evolution was repeated once with similar results. Red, unmutated residue in TR, lilac, mutations affecting the SS; purple; mutations characteristic of variant 1.1; green; mutations characteristic of variant 3.2. Source data are provided as a Source Data file.

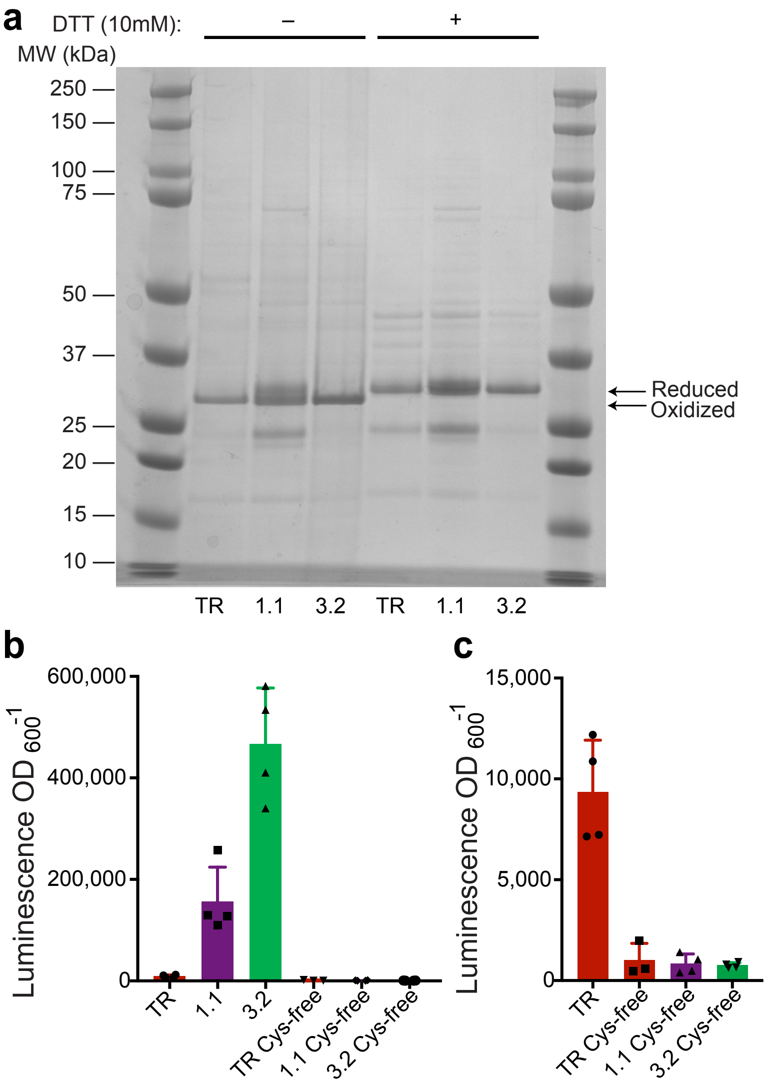

Supplementary Figure 12. Trastuzumab and evolved variants require disulfides for activity. (a) Coomassie gel showing purified trastuzumab (TR) scFv and evolved variants 1.1 and 3.2 with or without the addition of dithiothretiol (DTT) as a reducing agent. (b–c) Luminescence-based transcriptional activation assay showing impact of removing disulfides from trastuzumab and evolved variants by mutating four disulfide-forming Cys residues to Ser. Bars represent mean and s.d. from four biological replicates pooled from two separate experiments. Variants 1.1 and 3.2 have been removed in (c) to allow lower values to be compared. Background or non-target bands in this figure are discussed in Supplementary Discussion. Source data are provided as a Source Data file.

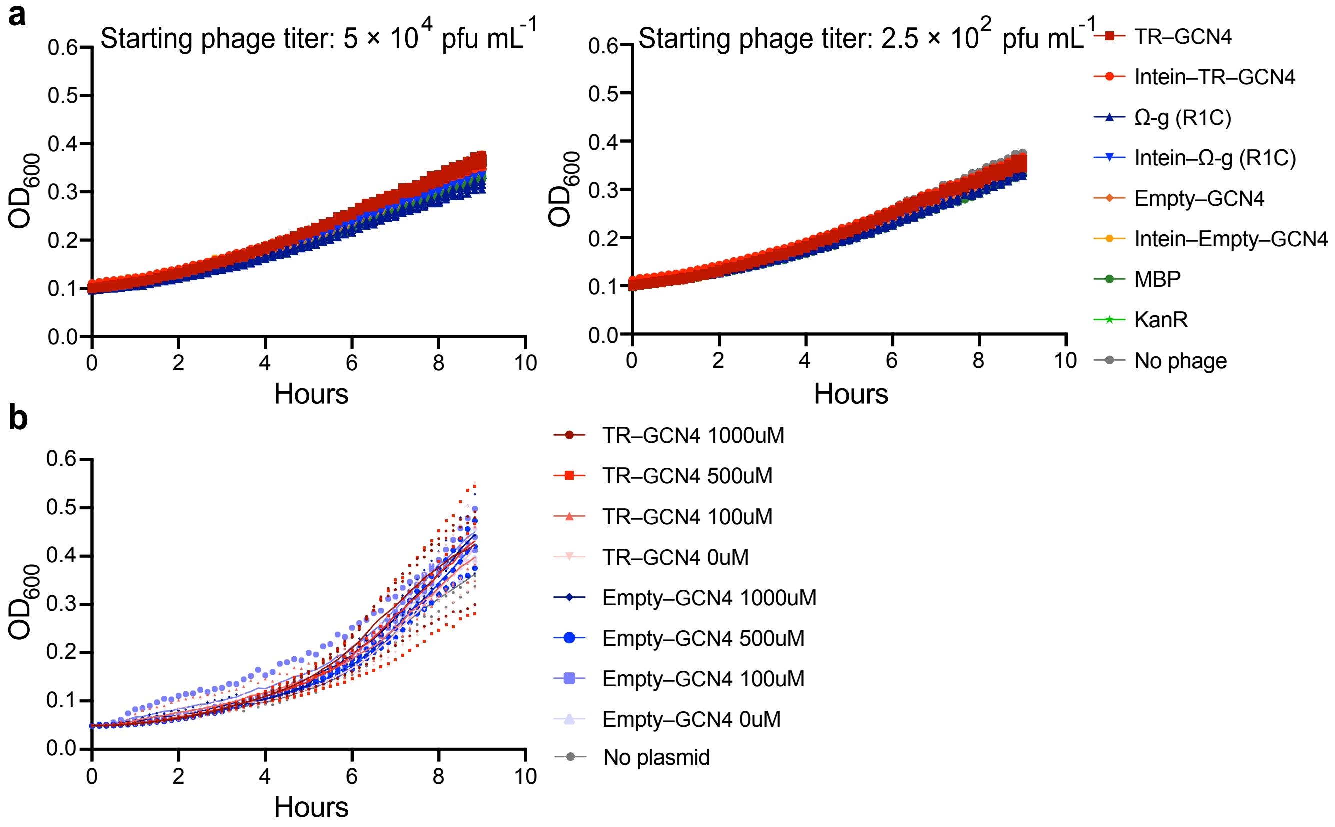

Supplementary Figure 13. Phage-based and plasmid-based periplasmic scFv expression does not impair host cell growth rate. (a) Timecourse growth assay measuring OD_{600} of host cells transformed with accessory plasmid pJC175e[6], which provides free pIII and allows selection-independent phage propagation, grown in the presence of two initial titers of selection or control phage. Three biological replicates are shown. (b) Timecourse growth assay measuring OD_{600} of host cells with plasmid-based expression of trastuzumab scFv under an arabinose-driven promoter. Arabinose concentrations are indicated in legend, right. Three biological replicates are shown. Points represent individual data, while lines indicate mean values. Source data are provided as a Source Data file.

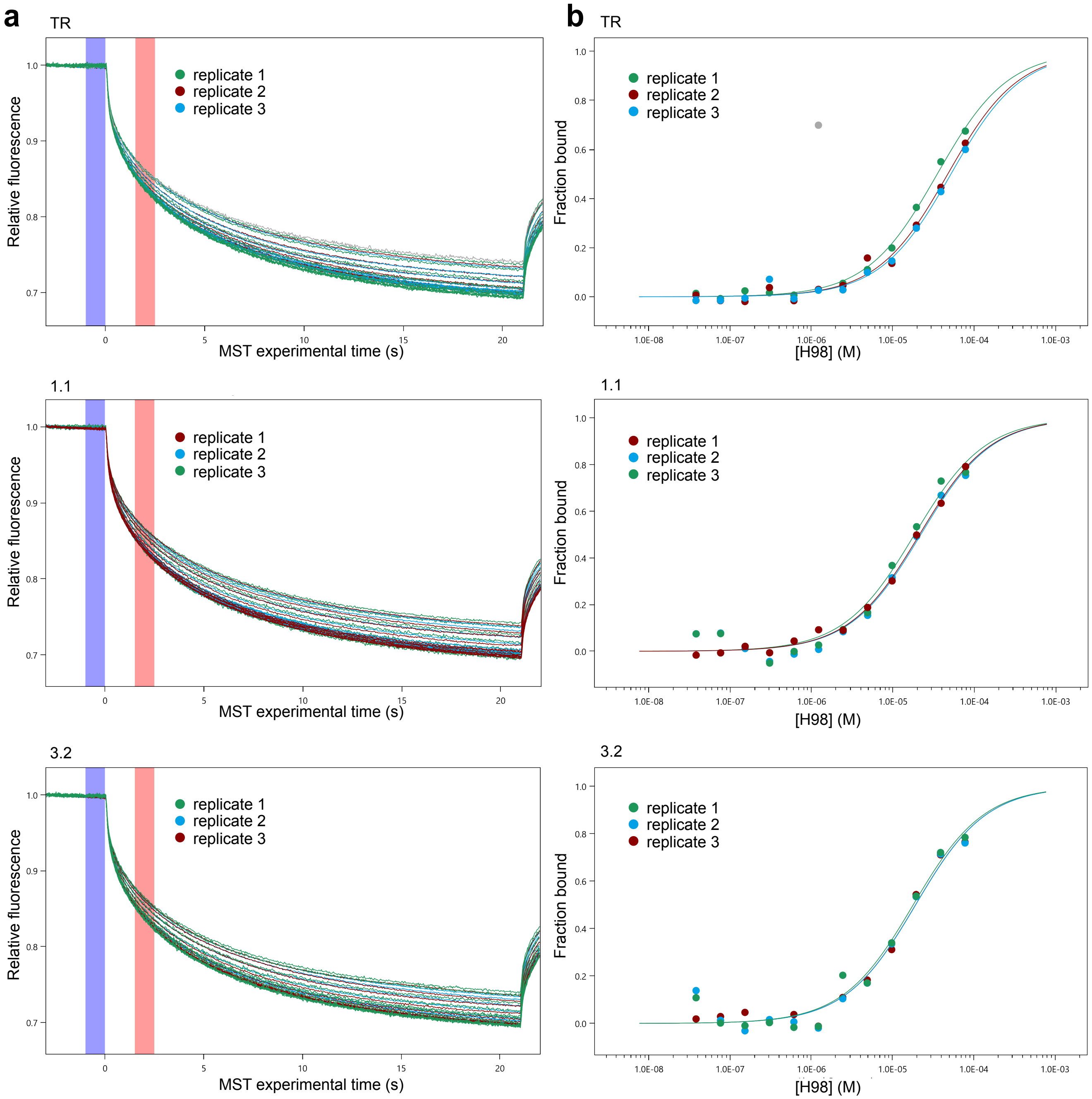

Supplementary Figure 14. Microscale thermophoresis analysis of trastuzumab scFv (TR) and variants 1.1 and 3.2. (a) MST raw data traces representing three technical replicates per sample. (b) Binding curves and individual data points for all replicates as calculated by MO.Affinity Analysis software version 3.2 (NanoTemper). One TR data point, shown in grey, was omitted from the analysis as an outlier due to evidence of fluorophore adsorption. Source data are provided as a Source Data file.

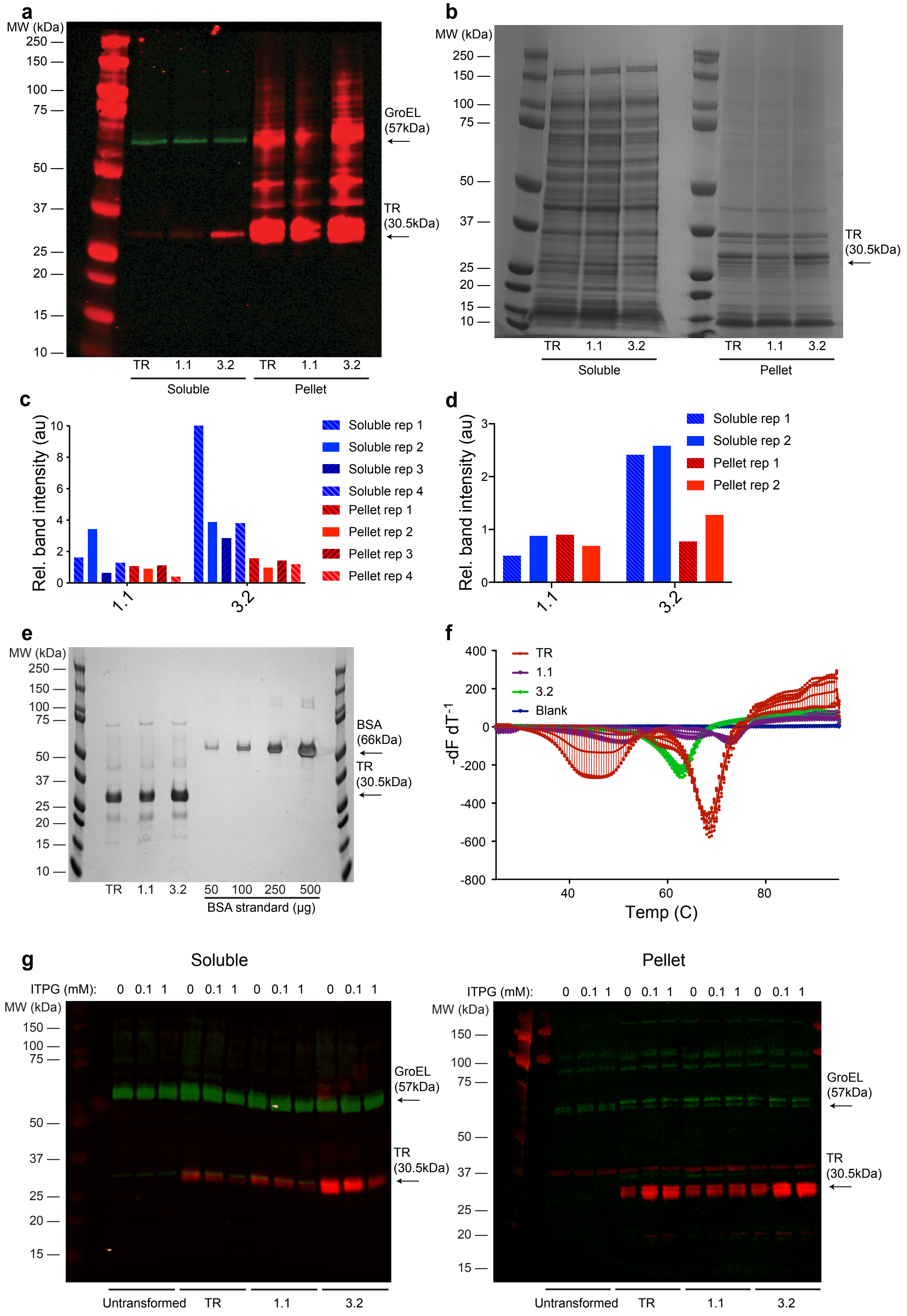
a
MW (kDa)
250
150
100
75
50
37
25
20
15
10
GroEL (57kDa)
TR (30.5kDa)
TR 1.1 3.2 TR 1.1 3.2
Soluble
Pellet
b
MW (kDa)
250
150
100
75
50
37
25
20
15
10
TR (30.5kDa)
TR 1.1 3.2 TR 1.1 3.2
Soluble
Pellet
c
Rel. band intensity (au)
Soluble rep 1
Soluble rep 2
Soluble rep 3
Soluble rep 4
Pellet rep 1
Pellet rep 2
Pellet rep 3
Pellet rep 4
1.1
3.2
d
Rel. band intensity (au)
Soluble rep 1
Soluble rep 2
Pellet rep 1
Pellet rep 2
1.1
3.2
e
MW (kDa)
250
150
100
75
50
37
25
20
15
10
BSA (66kDa)
TR (30.5kDa)
TR 1.1 3.2 50 100 250 500
BSA strandard (µg)
f
TR
1.1
3.2
Blank
-dF dT-1
Temp (C)
g
Soluble
Pellet
ITPG (mM): 0 0.1 1 0 0.1 1 0 0.1 1 0 0.1 1
MW (kDa)
150
100
75
50
37
25
20
15
GroEL (57kDa)
TR (30.5kDa)
Untransformed TR 1.1 3.2

Supplementary Figure 15. Soluble expression and thermostability characteristics of evolved trastuzumab variants 1.1 and 3.2. (a–b) Full western blot and Coomassie gel of TR and evolved variants expressed from P_{T7Lac} in BL21*DE3 cells, shown in main text Fig. 5g. Red channel: anti-6XH. Green channel: anti-GroEL. (c–d) Relative expression levels of trastuzumab variants 1.1 and 3.2 as determined by gel densitometry in western blotting (c) and in Coomassie-stained SDS-PAGE gel (d). Band intensities are normalized first to a reference band, then to band intensity of unmodified trastuzumab. Two or more replicate experiments conducted on separate days and with fresh transformations of BL21*DE3 cells are shown for each. (e) SDS-PAGE of purified trastuzumab (TR) and variants 1.1 and 3.2 expressed in BL21*DE3 cells at 16 °C. A BSA standard is also shown. These samples were used in diluted form in representative ELISA and MST data (Fig. 5f, Table 1, Supplementary Fig. 1b-c). This assay was repeated once with similar results. The Coomassie-stained SDS-PAGE gel showing diluted samples can be found in Supplementary Fig. 16b. (f) Melting temperature curves of trastuzumab scFv and evolved variants. Data reflects individual data points, mean and s.d. of pooled data from experiments conducted with separate protein preps and on separate days, each with four technical replicates. Purified protein used in both replicates can be found in Supplementary Fig. 15c. (g) Additional western blot showing two levels of expression of TR and evolved variants from the IPTG-inducible T_{7Lac} promoter in BL21*DE3 cells, as well as untransformed controls. Background or non-target bands in this figure are discussed in Supplementary Discussion. This assay was repeated for a total of four biological replicates for soluble fractions and two biological replicates for insoluble fractions. Source data are provided as a Source Data file.

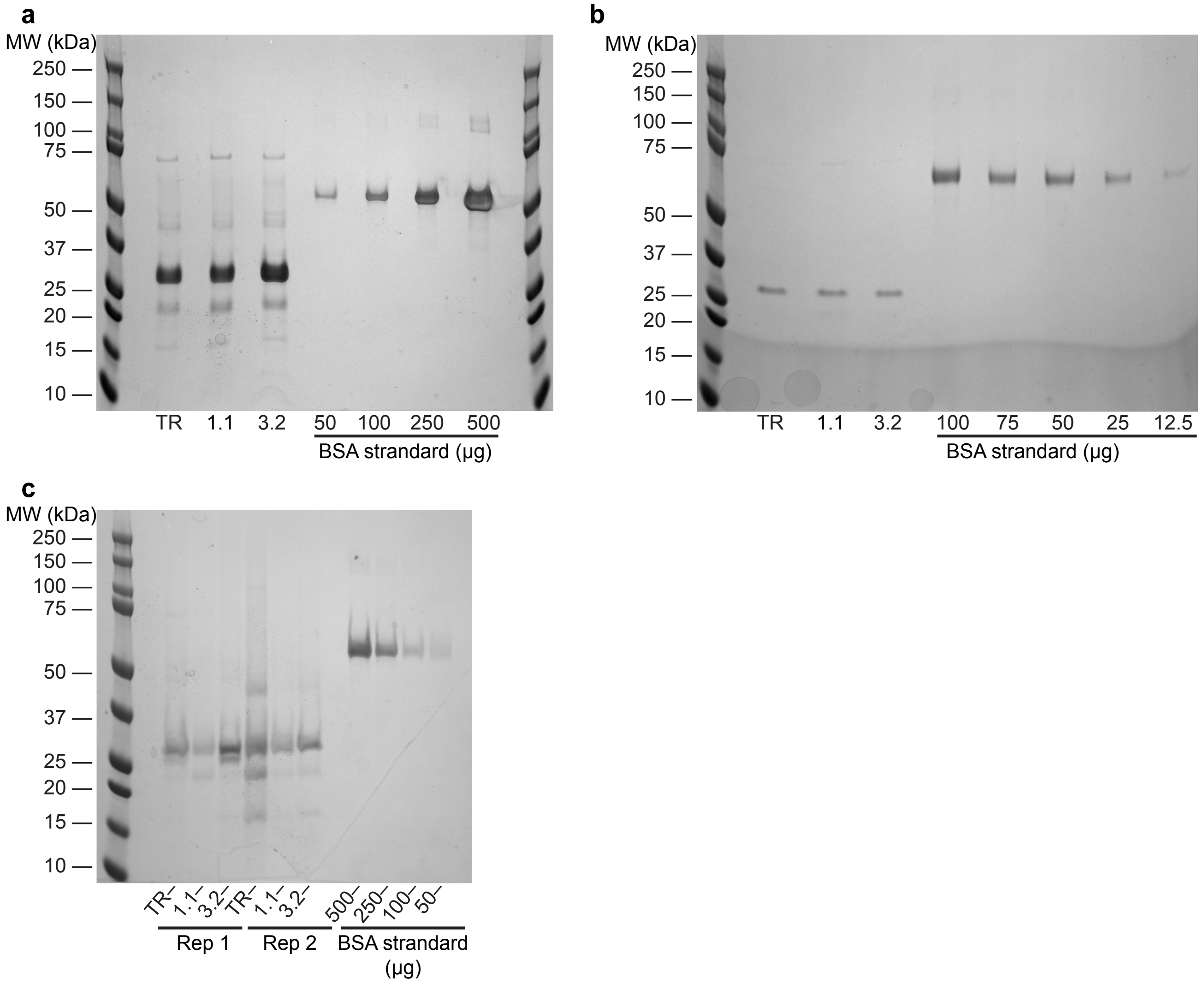

Supplementary Figure 16. Trastuzumab scFv and evolved variants used in biochemical characterizations. (a) Initial protein purification and (b) 25 µg/mL dilution of trastuzumab scFv and variants 1.1 and 3.2 used in MST and representative ELISA experiments (Fig. 5f, Table 1, Supplementary Fig. 4, Supplementary Fig. 1b-c). (a) is identical to Supplementary Fig. 6e; shown again here for comparison. (c) Two replicate protein purifications used in thermal melt experiments (Table 1, Supplementary Fig. 6f). BSA standards also shown. Background or non-target bands in this figure are discussed in Supplementary Discussion. Gels (a-c) were reproduced once with similar results. Source data are provided as a Source Data file.

Supplementary Table 1. Plasmid names, strains, phage and arabinose induction concentrations used in this work.

Fig.	Strain	Plasmids	Phage	Ara (µM)
2b	S2060[7]	pAR26c2 pAR27a/pAR27b/pAR27c/pAR27d		80
2c	S536	pMM102b2	SP53a/SP53a2/SP53a3	
2d	S536	pMM102b2 DP6[8]	SP53a2	
2e	BL21*DE3	pMM130y1/y2/y3		
2f	S536	pAR26c2 pMM103a/pMM103b/pMM103g/pMM103i		333.3
3b	S2060	pAR26c2 pTW200a1/pTW200b1 +/-pMM95a		12.3
3c	S536	pMM102g1 pMM95g7 DP6	SP30b1	
3d	S536	pAR26c2 pMM95h4 pMM109d1/pMM109d2/pMM109d3/ pMM109d4/pMM109d5		333.3
4b	S1367	pMM116f pMM114a	37o5c2.1/40o4c4.2/ 37o5c/40o4c/37o5c-0	
4c	S1367	pMM116f pMM114a	SP40o4c/SP40o4c4.2	
4e	S1367	pMM116f pMM114a DP6	SP37o5c-0	
4f	S1367	pMM116f pMM114a DP6	SP40o4c	
4g	S1367	pMM116f pMM114a DP6	SP37o5c-0/SP40o4c	
4h	S536	pMM96z pMM95h4 pMM109d1/d2/d5		333.3
4i	BL21* DE3	pMM130o1/pMM130o1b		
5b	S536	pMM116e2 pMM119d4b	SP37d4/SP40d1/SP37z4 /SP40z1	
5c–d	S536	pMM116f pMM119d4b DP6	SP37d4/SP40d1	
5e	S536	pMM96z pMM119d4b pMM129d1/pMM129d1g/ pMM129d1h		111.1
5f,g, h	BL21* DE3	pMM130c1/pMM130c1g/pMM130c1h		
S1b	S2060	pTW161a/pTW161a2 pTW160a/pTW160b/pTW160c		
S2b	S2060	pAR26c2	SP53a/SP53a2/M13	
S2c	S536	pAR26c2	SP53a/SP53a2/SP53a3/ M13	
S3	S536	pMM102b2 DP6	SP53a2	
S4a	S536	pAR26c2 pAR27a/pAR27c		
S4a	BL21*DE3	pMM130y1/pMM130y2/pMM130y3		
S4d-e	S536	pAR26c2 pAR27a/pAR27c/pMM103g		
S5a	S536	pMM102g1 pMM95g7 for PACE, pMM102g2 pMM95g7 for PANCE	SP30a1/SP30b1	
S5b	S536	pMM102g1 pMM95g7 DP6	SP30b1	
S6a	S536	pAR26c2 pMM95a pTW200a1/pMM109a1/ pMM109a2/pMM109a3/pMM109a4		37
S6c	S536	pMM95g7 pMM116a1	SP30a1/SP31	
S6d	S536	pMM102b2 pMM95a/pMM95d	SP30a1	37
S6g-h	S1367	pMM150a1 pMM130o1/pMM130o5		Var-ious
S7a	S536	pMM116e2 pMM119d4b	SP37d3/SP37z1/ SP37d4/SP37z4	

S8a–d	S1367	pMM114a1 pMM116a/pMM116e2/pMM116f/pMM116g	SP37o5c/SP37o5c2SP37o5c2.1/ SP40o4c/SP40o4c4.2	
S8e	S536	pMM96z pMM114a pMM129o1/pMM129o2/pMM129o3/pMM129o4		37
S8f	S1367	pMM114a1 pMM116a/pMM116e2/pMM116f/pMM116g	SP37o5c	
S9a	S1367	pMM114a pMM116f DP6	SP37o5c-0	
S9b	S1367	pMM114a pMM116f DP6	SP40o4c	
S9c	BL21* DE3	pMM130o1/pMM130o1b		
S10a	S536	pMM116f pMM119d4b DP6	SP37d4/SP40d1	
S10b–c	BL21* DE3	pMM130c1/pMM130c1g/pMM130c1h		
S11b-c	S536	pMM116g pMM119d6b DP6	SP37d4/SP40d1 120 h populations L1, L3	
S12a	BL21* DE3	pMM130c1/pMM130c1g/pMM130c1h		
S12b–c	S536	pMM96z pMM119d4b pMM129d1/pMM129d1g/ pMM129d1h/pMM129d3/pMM129d3g/pMM129d3h		
S13ab	S1367	pJC175e[6]	SP37d4/SP40d1/SP37o5c/SP40o4c/SP37z1/SP40z1/SP01a[9]/SP13[9]	
S113c	S1367	pJC175e[6] pMM129d2/pMM129z2		Var-ious
S14	BL21* DE3	pMM130c1/pMM130c1g/pMM130c1h		
S15	BL21* DE3	pMM130c1/pMM130c1g/pMM130c1h		
S16a-c	BL21* DE3	pMM130c1/pMM130c1g/pMM130c1h		

Supplementary Table 2. Plasmids used in this work. CP: complementary plasmid. A complementary plasmid takes the place of an evolving selection phage in plasmid-based assays such as transcription activation assays.

Name	Class (resistance)	Ori	Prom	[RBS][4] Genes	Prom	[RBS] Genes
DP6	MP (chlorR)	cloDF-13	P_{BAD}	dnaQ926, dam, seqA, emrR, ugi, cda1	P_c	araC
			$P_{psp-tet}$	[sd8] gIII		
pAR26c2	AP1 (carbR)	SC101	P_{pro3}[10]	[SD8] $CadC_{1–155}$-Leu(16)TM[11]–HA4	P_{CadBA}	[sd8] luxAB
pAR27a	CP (specR)	ColE1	P_{BAD}	[sd8] SS–YibK–SH2	P_c	araC
pAR27b	CP (specR)	ColE1	P_{BAD}	[sd8] YibK–SH2	P_c	araC
pAR27c	CP (specR)	ColE1	P_{BAD}	[sd8] SS–YibK(V139R)–SH2	P_c	araC
pAR27d	CP (specR)	ColE1	P_{BAD}	[sd8] YibK(V139R) –SH2	P_c	araC
pMM95a	AP2 (chlorR)	p15A	P_{BAD}	[sd8] SS_{PelB}–GCN4(7P14P)–SH2	P_c	araC
pMM95d	AP2 (chlorR)	p15A	P_{BAD}	[sd8] SS_{PelB}–GCN4(7P14P)–SH2	P_{pro3}	[SD8] $SS_{1–8}$–NpuN
			P_c	araC		
pMM95g7	AP2 (chlorR)	p15A	P_{pro3}	[sd5] $CadC_{1–155}$–Leu(16)TM–GCN4(7P14P)	P_{pro3}	[SD8] $SS_{1–8}$–NpuN
pMM95h4	AP2 (chlorR)	p15A	P_{BAD}	[sd5] $CadC_{1–155}$–Leu(16)TM–GCN4(7P14P)	P_{pro3}	[SD8] $SS_{1–8}$–NpuN
			Pc	araC		
pMM96z	AP1 (carbR)	SC101	P_{CadBA}	[sd8] luxAB		
pMM102b2	AP1 (carbR)	SC101	P_{pro1}	[sd8] $CadC_{1–155}$–Leu(16)TM–HA4	P_{CadBA}	[sd8] gIII
pMM102g1	AP1 (carbR)	SC101	P_{pro1}	[sd6] $CadC_{1–155}$–Leu(16)TM–HA4	P_{CadBA}	[sd8] gIII
pMM102g2	AP1 (carbR)	SC101	P_{pro1}	[sd5] $CadC_{1–155}$–Leu(16)TM–HA4	P_{CadBA}	[sd8] gIII
pMM102g7	AP1 (carbR)	SC101	P_{pro1}	[sd4U] $CadC_{1–155}$–Leu(16)TM–HA4	P_{CadBA}	[sd5] gIII
pMM103a	CP (specR)	ColE1	P_{BAD}	[sd8] SS–YibK–SH2	P_c	araC
pMM103b	CP (specR)	ColE1	P_{BAD}	[sd8] SS–YibK(V139R)–SH2	P_c	araC
pMM103g	CP (specR)	ColE1	P_{BAD}	[sd8] SS–YibK(V139R R146C)–SH2	P_c	araC
pMM103i	CP (specR)	ColE1	P_{BAD}	[sd8] SS–YibK(V139R A138D)–SH2	P_c	araC
pMM109a1	CP (specR)	ColE1	P_{pro1}	[sd5] $SS_{8–CNF–9–}$ Ω-graft-SH2		
pMM109a2	CP (specR)	ColE1	P_{pro1}	[sd5] $SS_{9–CNF–10–}$ Ω-graft-SH2		
pMM109a3	CP (specR)	ColE1	P_{pro1}	[sd5] $SS_{10–CNF–11–}$ Ω-graft–SH2		
pMM109a4	CP (specR)	ColE1	P_{pro1}	[sd5] $SS_{11–CNF–12–}$ Ω-graft–SH2		
pMM109d1	CP (specR)	ColE1	P_{pro3}	[sd5] NpuC–$SS_{9–21}$–Ω-graft–SH2		
pMM109d2	CP (specR)	ColE1	P_{pro3}	[sd5] NpuC–$SS_{9–20}$–Ω-graft(L231F 232A)-SH2		
pMM109d3	CP (specR)	ColE1	P_{pro3}	[sd5] NpuC–$SS_{9–20}$–Ω-graft(L224S L231F F232A)-SH2		
pMM109d4	CP (specR)	ColE1	P_{pro3}	[sd5] NpuC–$SS_{9–20}$–Ω-graft(R1C L231F F232A)-SH2		
pMM109d5	CP (specR)	ColE1	P_{pro3}	[sd5] NpuC–$SS_{9–20}$–Ω-graft(G119C L231F F232A)-SH2		
pMM114a	AP2 (specR)	p15A	P_{pro3}	[sd5] $CadC_{1–155}$–Leu(16)TM–GCN4(7P14P)	P_{pro3}	[SD8] $SS_{1–8}$–NpuN

pMM116a	AP1 (carbR)	SC101	P_{cadBA}	[sd8] gIII		
pMM116e2	AP1 (carbR)	SC101	P_{cadBA}	[sd6] gIII, luxAB		
pMM116f	AP1 (carbR)	SC101	P_{cadBA}	[sd2] gIII		
pMM116g	AP1 (carbR)	SC101	P_{cadBA}	[sd2G] gIII		
pMM119d4b	AP2 (specR)	ColE1	P_{pro3}	[sd5] CadC$_{1–155}$–Leu(16)TM–H98	P_{pro3}	[SD8] SS$_{1–8}$–NpuN
pMM119d6b	AP2 (specR)	ColE1	P_{pro1}	[sd5] CadC$_{1–155}$–Leu(16)TM–H98	P_{pro3}	[SD8] SS$_{1–8}$–NpuN
pMM129d1	CP (chlorR)	p15A	P_{BAD}	[sd8] SS–trastuzumab–YibK	P_c	araC
pMM129d1g	CP (chlorR)	p15A	P_{BAD}	[sd8] SS–trastuzumab(H91Y)–YibK	P_c	araC
pMM129d1h	CP (chlorR)	p15A	P_{BAD}	[sd8] SS–trastuzumab(A34D Y49S)–YibK	P_c	araC
pMM129d2	CP (chlorR)	p15A	P_{BAD}	[sd8] SS–trastuzumab–GCN4	P_c	araC
pMM129d3	CP (chlorR)	p15A	P_{BAD}	[sd8] SS–trastuzumab(C23S C88S C150S C224S)–YibK	P_c	araC
pMM129d3g	CP (chlorR)	p15A	P_{BAD}	[sd8] SS–trastuzumab(C23S C88S H91Y C150S C224S)–YibK	P_c	araC
pMM129d3h	CP (chlorR)	p15A	P_{BAD}	[sd8] SS–trastuzumab(C23S A34D Y49S C88S C150S C224S)–YibK	P_c	araC
pMM129o1	CP (chlorR)	p15A	P_{BAD}	[sd8] SS–Ω-graft–YibK	P_c	araC
pMM129o2	CP (chlorR)	p15A	P_{BAD}	[sd8] SS–Ω-graft(L231F)–YibK	P_c	araC
pMM129o3	CP (chlorR)	p15A	P_{BAD}	[sd8] SS–Ω-graft(F232A)–YibK	P_c	araC
pMM129o4	CP (chlorR)	p15A	P_{BAD}	[sd8] SS–Ω-graft(231F 232A)–YibK	P_c	araC
pMM129z2	CP (chlorR)	p15A	P_{BAD}	[sd8] SS–GCN4	P_c	araC
pMM130c1	EP (kanR)	pBR322	P_{T7lac}	SS–trastuzumab–6XHis		
pMM130c1g	EP (kanR)	pBR323	P_{T7lac}	SS–trastuzumab(91Y) –6XHis		
pMM130c1h	EP (kanR)	pBR323	P_{T7lac}	SS–trastuzumab(34D 49S) –6XHis		
pMM130o1	EP (kanR)	pBR322	P_{T7lac}	SS–Ω-graft–6XHis		
pMM130o1b	EP (kanR)	pBR323	P_{T7lac}	SS–Ω-graft(224S)–6XHis		
pMM130o5	EP (kanR)	pBR323	P_{T7lac}	NpuC–SS$_{9–20}$–Ω-graft–6XHis		
pMM130y1	EP (kanR)	pBR323	P_{T7lac}	SS–YibK –6XHis		
pMM130y2	EP (kanR)	pBR322	P_{T7lac}	SS–YibK(V139R)–6XHis		
pMM130y3	EP (kanR)	pBR323	P_{T7lac}	SS–YibK(V139R R146C)–6XHis		
pMM150a1	CP (specR)	ColE1	P_{BAD}	[sd5] SS$_{1–8}$–NpuN	P_c	araC
pTW161a	CP (specR)	ColE1	P_{BAD}	[sd8] CadC$_{1–155}$–Leu(16)TM–GCN4	P_c	araC
pTW161a2	CP (specR)	ColE1	P_{BAD}	[sd8] CadC$_{1–155}$–Leu(16)TM–GCN4(7P14P)	P_c	araC
pTW160a	AP1 (carbR)	SC101	P_{cadBA}	luxAB		
pTW160b	AP1 (carbR)	SC101	$P_{cadBA\ (-427–+1)}$	luxAB		
pTW160ac	AP1 (carbR)	SC101	$P_{cadBA\ (-288–+1)}$	luxAB		
pTW200a1	AP2 (specR)	ColE1	P_{pro1}	[sd5] SS–Ω-graft–SH2		
pTW200b1	AP2 (specR)	ColE2	P_{pro1}	[sd5] SS–Ω-graft(L231F F232A) –SH2		

Supplementary Table 3. Selection phage used in this work.

Name	Class	Ori	Prom	[RBS][4] Genes
M13	Ref.	M13 f1	P_{gIII}	gIII
SP01a[9]	SP	M13 f1	P_{gIII}	[RpoZ] MBP–T7N
SP13[9]	SP	M13 f1	P_{gIII}	[RpoZ] KanR
SP30a1	Selection phage (SP)	M13 f1	P_{gIII}	[sd8] NpuC–SS$_{9–20}$–Ω-graft–SH2
SP30b1	SP	M13 f1	P_{gIII}	[sd8] NpuC–SS$_{9–20}$–Ω-graft(L231F F232A) –SH2
SP31	SP	M13 f1	P_{gIII}	[sd8] SS$_{9–20}$–Ω-graft–SH2
SP35a1	SP	M13 f1	P_{gIII}	[sd8] NpuC–SS$_{9–20}$–Ω-graft(G119C)
SP35a2	SP	M13 f1	P_{gIII}	[sd8] NpuC–SP$_{9–20}$–Ω-graft(G119C L231F F232A)
SP37d3	SP	M13 f1	P_{gIII}	[sd8] SS–trastuzumab–YibK
SP37d4	SP	M13 f1	P_{gIII}	[sd8] SS–trastuzumab–GCN4
SP37o5c	SP	M13 f1	P_{gIII}	[sd8] SS–Ω-graft(R1C L231F F232A)
SP37o5c-0	SP	M13 f1	P_{gIII}	[sd8] SS–Ω-graft(R1C W100* L231F F232A)
SP37o5c2.1	SP	M13 f1	P_{gIII}	[sd8] SS–Ω-graft(R1C F232A)
SP37z1	SP	M13 f1	P_{gIII}	[sd8] SS–YibK
SP37z4	SP	M13 f1	P_{gIII}	[sd8] SS–GCN4
SP40d1	SP	M13 f1	P_{gIII}	[sd8] NpuC–SS$_{9–20}$–trastuzumab–GCN4
SP40o4c	SP	M13 f1	P_{gIII}	[sd8] NpuC–SS$_{9–20}$–Ω-graft(R1C L231F F232A)
SP40o4c4.2	SP	M13 f1	P_{gIII}	[sd8] NpuC–SS$_{9–20}$–Ω-graft(R1C L232A)
SP40z1	SP	M13 f1	P_{gIII}	[sd8] NpuC–SS$_{9–20}$–GCN4
SP53a	SP	M13 f1	P_{gIII}	[sd8] SS–YibK–SH2
SP53a2	SP	M13 f1	P_{gIII}	[sd8] SS–YibK(V139R)–SH2
SP53a3	SP	M13 f1	P_{gIII}	[sd8] SS–SH2

Supplementary Table 4. Primers used in this work.

AB1793	(5'–TAATGGAAACTTCCTCATGAAAAAGTCTTTAG)
AB1396	5'–ACAGAGAGAATAACATAAAAACAGGGAAGC)
AR007	(5'–ATGTTGAAAATCTCCTTCUAGATTA)
MM557	(5'– TGTGGCAATTATCATTGCATCATTCCCTTTTCGAATGAGTTTCTATTATGTGTAGGCTGGAGCTGCTTCG)
MM558	5'–AAAATAACGTCTTGCATTCACC)
MM559	5'– TGGCAAGCCACTTCCCTTGTACGAGCTAATTATTTTTTGCTTTCTTCTTTATTCCGGGGATCCGTCGACC)
MM560	(5'–TTCATGTGTTCTCCTTATGAGC)
MM1081	(5'–AGGTGGGGGTUCAGGC)
MM1082	(5'–AACCCCCACCUCCGGAG)
TW629	(5'–GACTCCCTGCAAGCCTCAG)
TW1243	(5'–CGAACCAGAAAAGAACCATTAATG)

Supplementary Table 5.

Properties of trastuzumab scFv and evolved variants determined by MST analysis. Values reflect mean and s.d. of three technical replicates in MST (Supplementary Fig. 14) and were calculated in MO.Affinity Analysis software, version 3.2 and Prism 8 sigmoidal 4-point log curve fitting. We note that MST data is not conclusive due to solubility limits of the H98 peptide, and is provided in support of ELISA data. Source data are provided as a Source Data file.

	EC_{50} (μM)	K_D (μM)
TR	4.3 ± 1.6	44.9 ± 8.7
1.1	1.4 ± 1.1	20.5 ± 1.9
3.2	1.3 ± 1.1	18.8 ± 0.7

Supplementary Discussion

Background banding

We note that background banding is visible in multiple gels and blots, most notably in Supplementary Fig. 4a and Supplementary Fig. d-e. These background bands appear in whole-cell lysates, are recognized by an anti-SH2 antibody, and are not visible in the uninduced control lanes, strongly suggesting that they represent partially proteolytically degraded forms of the YibK–SH2 construct which retain the anti-SH2 epitope recognized by the antibody. Similarly, background banding between 30kDa and 60kDa likely represents partially degraded forms of the dimeric species. A ~15kDa background band is visible in Supplementary Fig. 6g which is only apparent in the spheroplast fraction and is not present in the untransformed or positive control, and which likely represents a degradation product of the cytoplasmic intein–scFv–6XHis species which retains the 6XHis tag. Similarly, a ~18kDa background band can be observed in a western blot of soluble protein in Supplementary Fig. 9e, and is only present in treatments with Ω-graft variant 2.8. This band is not visible in other replicates (Supplementary Fig. 9c) and may be the result of overexpression. In Supplementary Figs. 9c, 9e and 15a, western blot lanes including the insoluble pellet fraction show background smearing that is not present in the untransformed or uninduced control lanes and likely represents aggregation of scFvs in the cytoplasmic environment. In Supplementary Fig. 15g, background bands are visible at ~31, ~36, ~80 and ~100kDa for all samples including untransformed controls, and likely represent off-target binding of the anti-6XHis and anti-GroEL antibodies.

In purified YibK, a ~45kDa background band is faintly visible by Coomassie (Supplementary Fig. 4b) staining but is not recognized by anti-6XHis antibody (Supplementary Fig. 4c), suggesting that it does not represent a YibK derivative but rather represents an *E. coli* protein contaminant. A potential candidate is the common, histidine-enriched *E. coli* IMAC contaminant ODO2 (44kDa). A 44kDa background banding can also be observed in purified protein in Supplementary Figs. 12 and 16[12]. Other background bands observed in these figures may represent ArnA (74kDa), SlyD (26kDa) and YodA (21kDa) common contaminants in IMAC purifications due to histidine-rich internal sequences[12,13].

Supplementary Note 1. DNA sequences used in this work.

a. Signal sequence (SS)

AAACAAAGCACTATTGCACTGGCACTCTTACCGTTACTGTTTACCCCTGTGACAAAAGCC

b. SS_{PelB}

AAATACCTGCTGCCGACCGCTGCTGCTGGTCTGCTGCTCCTCGCTGCCCAACCGGCAA
TGGCC

c. -600– +1 P_{CadBA}[14]

TGCCGGAATTGAACAACCTGTCCATTATATATCAAATAAAAGCGGTCAGTGCTCTGGTAA
AAGGTAAAACAGATGAGTCTTACCAGGCGATAAATACTGGCATTGATCTTGAAATGTCCT
GGCTAAATTATGTGTTGCTTGGCAAGGTTTATGAAATGAAGGGGATGAACCGGGAAGCA
GCTGATGCATATCTCACCGCCTTTAATTTACGCCCAGGGGCAAACACCCTTTACTGGATT
GAAAATGGTATATTCCAGACTTCTGTTCCTTATGTTGTACCTTATCTCGACAAATTTCTTG
CTTCAGAATAAGTAACTCCGGGTTGATTTATGCTCGGAAATATTTGTTGTTGAGTTTTTGT
ATGTTCCTGTTGGTATAATATGTTGCGGCAATTTATTTGCCGCATAATTTTTATTACATAAA
TTTAACCAGAGAATGTCACGCAATCCATTGTAAACATTAAATGTTTATCTTTTCATGATAT
CAACTTGCGATCCTGATGTGTTAATAAAAAACCTCAAGTTCTCACTTACAGAAACTTTTGT
GTTATTTCACCTAATCTTTAGGATTAATCCTTTTTTCGTGAGTAATCTTATCGCCA

d. $CadC_{1–155}$[11]

ATGCAACAACCTGTAGTTCGCGTTGGCGAATGGCTTGTTACTCCGTCCATAAACCAAATT
AGCCGCAATGGGCGTCAACTTACCCTTGAGCCGAGATTAATCGATCTTCTGGTTTTCTTT
GCTCAACACAGTGGCGAAGTACTTAGCAGGGATGAACTTATCGATAATGTCTGGAAGAG
AAGTATTGTCACCAATCACGTTGTGACGCAGAGTATCTCAGAACTACGTAAGTCATTAAA
AGATAATGATGAAGATAGTCCTGTCTATATCGCTACTGTACCAAAGCGCGGCTATAAATT
AATGGTGCCGGTTATCTGGTACAGCGAAGAAGAGGGAGAGGAAATAATGCTATCTTCGC
CTCCCCCTATACCAGAGGCGGTTCCTGCCACAGATTCTCCCTCCCACAGTCTTAACATT
CAAAACACCGCAACGCCACCTGAACAATCCCCAGTTAAAAGCAAACGA

e. Leu(16)TM[11]

GGCGGCCCAGGGTTACTGCTGTTACTGCTACTTCTTTTATTGTTATTACTGTTGTTATTG
GGTCCAGGTGGC

f. HA4

GGCAGCTCTGTGAGTAGCGTTCCGACCAAACTGGAAGTGGTTGCAGCAACCCCGACGA
GCCTGCTGATTTCTTGGGATGCCCCGATGTCTAGTAGCTCTGTGTATTACTATCGTATCA
CCTACGGTGAAACGGGCGGTAACAGCCCGGTGCAGGAATTTACGGTTCCGTATAGTAG
CTCTACCGCGACGATTAGTGGCCTGAGCCCGGGTGTGGATTACACCATCACGGTTTATG
CATGGGGCGAAGATAGCGCGGGTTACATGTTCATGTATTCTCCGATTAGTATCAATTATC
GTACCTGC

g. SH2

AGTCTGGAAAAACACAGCTGGTATCATGGCCCTGTGAGCCGTAACGCGGCCGAATACCT
GCTGAGCTCTGGCATTAATGGTTCTTTTCTGGTTCGTGAAAGTGAAAGTAGCCCGGGCC
AGCGCAGCATTTCTCTGCGTTATGAAGGTCGCGTGTATCACTACCGTATCAACACCGCC
AGCGATGGCAAACTGTACGTTTCTAGTGAATCTCGCTTCAATACCCTGGCAGAACTGGT
GCATCACCATAGCACGGTTGCGGATGGTCTGATCACCACGCTGCATTATCCGGCGCCG
AAACGC

h. YibK. Position 139 is shown in red.

ATGCTGGACATTGTCTTGTACGAACCTGAAATTCCGCAGAACACGGGCAACATCATTCG
TTTGTGTGCAAACACAGGATTTCGTCTTCACTTAATCGAGCCGCTGGGGTTCACTTGGG
ATGACAAACGCCTTCGCCGTTCCGGGTTGGATTACCACGAGTTCGCCGAAATTAAACGC
CACAAAACCTTTGAGGCTTTTCTGGAGAGCGAGAAACCTAAACGTTTGTTTGCCCTTACC
ACCAAGGGATGCCCCGCTCATTCGCAAGTAAAGTTTAAATTAGGGGATTACCTGATGTTC
GGCCCAGAGACACGCGGAATTCCCATGTCGATTCTTAATGAAATGCCGATGGAACAGAA
GATCCGCATTCCGATGACCGCGAACTCGCGTTCCATGAACCTTAGCAATTCTGTCGCCG
TGACAGTCTATGAGGCTTGGCGTCAATTAGGATATAAGGGGGCAGTTAATCTGCCCGAG
GTGAAA

i. YibK variant 3.7. R139 is shown in red. PANCE mutations are shown in blue.

ATGCTGGACATTGTCTTGTACGAACCTGAAATTCCGCAGAACACGGGCAACATCATTCG
TTTGTGTGCAAACACAGGATTTCGTCTTCACTTAATCGAGCCGCTGGGGTTCACTTGGG
ATGACAAACGCCTTCGCCGTTCCGGGTTGGATTACCACGAGTTCGCCGAAATTAAACGC
CACAAAACCTTTGAGGCTTTTCTGGAGAGCGAGAAACCTAAACGTTTGTTTGCCCTTACC
ACCAAGGGATGCCCCGCTCATTCGCAAGTAAAGTTTAAATTAGGGGATTATCTGATGTTC
GGCCCAGAGACACGCGGAATTCCCATGTCGATTCTTAATGAAATGCCGATGGAACAGAA
GATCCGCATTCCGATGACCGCGAACTCGCGTTCCATGAACCTTAGCAATTCTGTCGATA
GGACAGTCTATGAGGATTGGTGTCAATTAGGATATAAGGGGGCAGTTAATCTGCCCGAG
GTGAAA

j. GCN4

TTGCAAAGAATGAAACAACTTGAAGACAAGGTTGAAGAATTGCTTTCGAAAAATTATCAC
TTGGAAAATGAGGTTGCCAGATTAAAGAAATTAGTTGGCGAACGC

k. GCN4(7P14P)

TTGCAAAGAATGAAACAACTTGAACCGAAGGTTGAAGAATTGCTTCCGAAAAATTATCAC
TTGGAAAATGAGGTTGCCAGATTAAAGAAATTAGTTGGCGAACGC

l. NpuN- SS_{1-8}

AAACAAAGCACTATTGCACTGTGTCTCAGCTACGAAACCGAAATCTTGACCGTCGAATAT
GGTCTGCTGCCAATCGGCAAGATTGTTGAAAAACGTATTGAATGTACGGTCTACTCAGT
GGATAACAACGGCAATATCTACACCCAGCCGGTGGCCCAGTGGCATGACCGTGGTGAA
CAGGAAGTGTTCGAATATTGTCTGGAAGACGGATCTTTAATCCGTGCCACAAAGGATCA

CAAATTTATGACTGTAGATGGTCAGATGCTCCCAATCGACGAAATTTTTGAACGCGAATT
AGACCTGATGCGCGTGGATAATCTCCCGAAT

m. NpuC–$SS_{9\text{-}20}$. $SS_{9\text{-}20}$ is shown in purple.

ATGATCAAAATTGCCACGCGTAAATATTTAGGCAAACAGAATGTTTATGATATCGGTGTC
GAGCGCGATCATAATTTCGCGCTGAAAAACGGCTTTATCGCCAGCAATTGTTTTAATGCA
CTCTTACCGTTACTGTTTACCCCTGTGACTAAAGCC

n. Ω-graft scFv. Positions 231 and 232 are shown in red. Position 100 is shown in green.

CGCGACATTGTTATGACGCAGTCGCCATCAAGCTTATCAGCGTCAGTGGGAGATCGCGT
TACAATTACATGCCGTTCGAGCACTGGGGCAGTCACAACCAGTAATTACGCTTCGTGGG
TCCAGGAAAAACCGGGTAAGTTGTTCAAGGGTTTGATTGGCGGTACTAATAACCGCGCA
CCGGGCGTCCCTAGCCGTTTTTCGGGGAGTTTGATTGGTGACAAGGCCACACTTACTAT
CAGCAGTCTGCAACCAGAGGATTTCGCTACATACTTTTGTGCATTGTGGTACTCCAACCA
TTGGGTCTTCGGTCAGGGCACGAAGGTTGAACTTAAACGCGGGGGTGGTGGCTCCGGA
GGTGGGGGTTCAGGCGGCGGAGGGTCTTCGGGTGGAGGGAGTGAGGTTAAGCTTCTT
GAAAGTGGTGGTGGTCTTGTGCAGCCTGGAGGCTCGTTAAAGCTGAGCTGCGCTGTGA
GTGGTTTCTCGTTGACGGATTATGGGGTCAATTGGGTACGCCAGGCACCGGGGCGTGG
CTTGGAGTGGATTGGCGTCATCTGGGGCGACGGAATCACTGATTATAACAGTGCCTTGA
AGGATCGCTTTATCATCAGCAAAGACGATTGCGAAAACACTGTCTATTTGCAAATGAGCA
AAGTTCGCTCGGATGATACGGCGTTATACTACTGTGTCACCGGACTTTTTGACTACTGG
GGGCAGGGCACTCTTGTCACGGTCTCCAGC

o. Ω-graft scFv variant 37o5c2.1. PACE mutations are shown in blue.

TGCGACATTGTTATGACGCAGTCGCCATCAAGCTTATCAGCGTCAGTGGGAGATCGCGT
TACAATTACATGCCGTTCGAGCACTGGGGCAGTCACAACCAGTAATTACGCTTCGTGGG
TCCAGGAAAAACCGGGTAAGTTGTTCAAGGGTTTGATTGGCGGTACTAATAACCGCGCA
CCGGGCGTCCCTAGCCGTTTTTCGGGGAGTTTGATTGGTGACAAGGCCACACTTACTAT
CAGCAGTCTGCAACCAGAGGATTTCGCTACATACTTTTGTGCATTGTGGTACTCCAACCA
TTGGGTCTTCGGTCAGGGCACGAAGGTTGAACTTAAACGCGGGGGTGGTGGCTCCGGA
GGTGGGGGTTCAGGCGGCGGAGGGTCTTCGGGTGGAGGGAGTGAGGTTAAGCTTCTT
GAAAGTGGTGGTGGTCTTGTGCAGCCTGGAGGCTCGTTAAAGCTGAGCTGCGCTGTGA
GTGGTTTCTCGTTGACGGATTATGGGGTCAATTGGGTACGCCAGGCACCGGGGCGTGG
CTTGGAGTGGATTGGCGTCATCTGGGGCGACGGAATCACTGATTATAATAGTGCCTTGA
AGGATCGCTTTATCATCAGCAAAGACGATTGCGAAAACACTGTCTATTTGCAAATGAGCA
AAGTTCGCTCGGATGATACGGCGTTATACTACTGTGTCACCGGACTCGCTGACTACTGG
GGGCAGGGCACTCTTGTCACGGTCTCCAGC

p. Ω-graft scFv variant 40o4c4.2. PACE mutations are shown in blue.

TGCGACATTGTTATGACGCAGTCGCCATCAAGCTTATCAGCGTCAGTGGGAGATCGCGT
TACAATTACATGCCGTTCGAGCACTGGGGCAGTCACAACCAGTAATTACGCTTCGTGGG
TCCAGGAAAAACCGGGTAAGTTGTTCAAGGGTTTGATTGGCGGTACTAATAACCGCGCA
CCGGGCGTCCCTAGCCGTTTTTCGGGGAGTTTGATTGGTGACAAGGCCACACTTACTAT
CAGCAGTCTGCAACCAGAGGATTTCGCTACATACTTTTGTGCATTGTGGTACTCCAACCA
TTGGGTCTTCGGTCAGGGCACGAAGGTTGAACTTAAACGCGGGGGTGGTGGCTCCGGA

GGTGGGGGTTCAGGCGGCGGAGGGTCTTCGGGTGGAGGGAGTGAGGTTAAGCTTCTT
GAAAGTGGTGGTGGTCTTGTGCAGCCTGGAGGCTCGTTAAAGCTGAGCTGCGCTGTGA
GTGGTTTCTCGTTGACGGATTATGGGGTCAATTGGGTACGCCAGGCACCGGGGCGTGG
CTTGGAGTGGATTGGCGTCATCTGGGGCGACGGAATCACTGATTATAACAGTGCCTTGA
AGGATCGCTTTATCATCAGCAAAGACGATTGCGAAAACACTGTCTATTTGCAAATGAGCA
AAGTTCGCTCGGATGATACGGCGTTATACTACTGTGTCACCGGATTAGCTGACTACTGG
GGGCAGGGCACTCTTGTCACGGTCTCCAGC

q. Ω-graft scFv variant 40o4c4.6. PACE mutations are shown in blue.

TGCGACAATGTTATGACGCAGTCGCCATCAAGCTTATCAGCGTCAGTGGGAGATCGCGT
TACAATTACATGCCGTTCGAGCACTGGGGCAGTCACAACCAGTAATTACGCTTCGTGGG
TCCAGGAAAAACCGGGTAAGGTGTTCAAGGGTTTGATTGGCGGTACTAATAACCGCGCA
CCGGGCGTCCCTAGCCGTTTTTCGGGGAGTTTGATTGGTGACAAGGCCACACTTACTAT
CAGCAGTCTGCAACCAGAGGATTTCGCTACATACTTTTGTGCATTGTGGTACTCCAACCA
TTGGGTCTTCGGTCAGGGCACGAAGGTTGAACTTAAACGCGGGGGTGGTGGCTCCGGA
GGTGGGGGTTCAGGCGGCGGAGGGTCTTCGGGTGGAGGGAGTGAGGTTAAGCTTCTT
GAAAGTGGTGGTGGTCTTGTGCAGCCTGGAGGCTCGTTAAAGCTGAGCTGCGCTGTGA
GTGGTTTCTCGTTGACGGATTATGGGGTCAATTGGGTACGCCAGGCACCGGGGCGTGG
CTTGGAGTGGATTGGCGTCATCTGGGGCGACGGAATCACTGATTATAACAGTGCCTTGA
AGGATCGCTTTATCATCAGCAAAGACGATTGCGAAAACACTGTCTATTTGCAAATGAGCA
AAGTTCGCTCGGATGATACGGCGTCATACTACTGTGTCACCGGATTCGCTGACTACTGG
GGGCAGGGCACTCTTGTCACGGTCTCCAGC

r. Ω-graft scFv variant 40o4c4.8. PACE mutations are shown in blue.

TGCGACATTGTTATGACGCAGTCGCCATCAAGCTTATCAGCGTCAGTGGGAGATCGCGT
TACAATTACATGCCGTTCGAGCACTGGGGCAGTCACAACCAGTAATTACGCTTCGTGGG
TCCAGGAAAAACCGGGTAAGTTGTTCAAGGGTTTGATTGGCGGTACTAATAACCGCGCA
CCGGGCGTCCCTAGCCGTTTTTCGGGGAGTTTGATTGGTGACAAGGCCACACTTACTAT
CAGCAGTCTGCAACCAGAGGATTTCGCTACATACTTTTGTGCATTGTGGTACTCCAACCA
TTGGGTCTTCGGTCAGGGCACGAAGGTTGAACTTAAACGCGGGGGTGGTGGCTCCGGA
GGTGGGGGTTCAGGCGGCGGAGGGTCTTCGGGTGGAGGGAGTGAGGTTAAGCTTCTT
GAAAGTGGTGGTGGTCTTGTGCAGCCTGGAGGCTCGTTAAAGCTGAGCTGCGCTGTGA
GTGGTTTCTCGTTGACGGATTATGGGGTCAATTGGGTACGCCAGGCACCGGGGCGTGG
CTTGGAGTGGATTGGCGTCATCTGGGGCGACGGAATCACTGATTATAACAGTGCCTTGA
AGGATCGCTTTATCATCAGCAAAGACGATTGCGAAAACACTGTCTATTTGCAAATGAGCA
AAGTTCGCTCGGATGATACGGCGTCATACTACTGTGTCACCGGATTAGCTGACTACTGG
GGGCAGGGCACTCTTGTCACGGTCTCCAGC

s. Trastuzumab scFv.

GACATTCAGATGACGCAGTCGCCATCAAGCTTAAGCGCCAGTGTGGGTGATCGCGTCA
CAATCACATGCCGTGCTTCCCAAGATGTAAATACCGCGGTGGCCTGGTATCAGCAAAAA
CCGGGAAAAGCTCCGAAGCTTTTAATTTACAGTGCATCGTTCCTTTATAGCGGGGTCCC
AAGCCGCTTTTCGGGTTCGCGCTCCGGGACCGACTTCACGCTTACGATTTCAAGCCTGC
AACCGGAAGATTTCGCCACATACTATTGCCAACAGCATTACACGACGCCGCCTACCTTC
GGGCAAGGCACGAAGGTGGAAATCAAACGCGGGGGAGGTGGCTCCGGAGGTGGGGGT
TCAGGCGGCGGAGGGTCTTCGGGTGGAGGGAGTGAGGTTCAGCTTGTGGAATCAGGT

GGAGGTTTAGTGCAACCTGGTGGTAGTTTACGCCTGTCCTGCGCAGCTAGTGGATTCAA
TATCAAAGACACTTATATCCATTGGGTACGTCAAGCCCCTGGGAAAGGACTGGAATGGG
TCGCCCGTATTTACCCCACTAACGGTTATACTCGTTACGCCGACTCTGTTAAGGGACGC
TTCACCATTAGTGCGGACACATCTAAAAACACAGCTTACTTGCAGATGAACTCCCTTCGT
GCAGAGGACACCGCCGTCTACTACTGTAGCCGTTGGGGAGGGGATGGATTTTATGCGA
TGGACTACTGGGGGCAGGGCACTCTTGTCACGGTCTCCAGC

t. SS–Trastuzumab scFv variant 1.1. SS is highlighted in purple. Mutated residues are shown in blue.

AAACAAAGCACTATTGCACTGGCACTCTTACCGTTACTGTTTATCCCTGTGACTAAAGCC
ATGCGGGACATTCAGATGACGCAGTCGCCATCAAGCTTAAGCGCCAGTGTGGGTGATC
GCGTCACAATCACATGCCGTGCTTCCCAAGATGTAAATACCGCGGTGGCCTGGTATCAG
CAAAAACCGGGAAAAGCTCCGAAGCTTTTAATTTACAGTGCATCGTTCCTTTATAGCGGG
GTCCCAAGCCGTTTTTCGGGTTCGCGCTCCGGGACCGACTTCACGCTTACGATTTCAAG
CCTGCAACCGGAAGATTTCGCCACATACTATTGCCAACAGTATTACACGACGCCGCCTA
CCTTCGGGCAAGGCACGAAGGTGGAAATCAAACGCGGGGGAGGTGGCTCCGGAGGTG
GGGGTTCAGGCGGCGGAGGGTCTTCGGGTGGAGGGAGTGAGGTTCAGCTTGTGGAAT
CAGGTGGAGGTTTAGTGCAACCTGGTGGTAGTTTACGCCTGTCCTGCGCAGCTAGTGG
ATTCAATATCAAAGACACTTATATCCATTGGGTACGTCAAGCCCCTGGGAAAGGACTGGA
ATGGGTCGCCCGTATTTACCCCACTAACGGTTATACTCGTTACGCCGACTCTGTTAAGG
GACGCTTCACCATTAGTGCGGACACATCTAAAAACACAGCTTACTTGCAGATGAACTCCC
TTCGTGCAGAGGACACCGCCGTCTACTACTGTAGCCGTTGGGGAGGGGATGGATTTTAT
GCGATGGACTACTGGGGGCAGGGCACTCTTGTCACGGTCTCCAGC

u. NpuC–SS_{9-20}–Trastuzumab scFv variant 3.2. SS_{9-20} is highlighted in purple. Mutated residues are shown in blue.

ATGATCAAAATTGCCACGCGTAAATATTTAGGCAAACAGAATGTTTATGATATCGGTGTC
GAGCGCGATCATAATTTCGCGCTGAAAAACGGCTTTATCGCCAGCAATTGTTTTAATGCA
CTCTTACCGTTACTGTTTACCCCTGTGACTAAAGACATGCGGGACATTCAGATGACGCA
GTCGCCATCAAGCTTAAGCGCCAGTGTGGGTGATCGCGTCACAATCACATGCCGTGCTT
CCCAAGATGTAAATACCGCGGTGGACTGGTATCAGCAAAAACCGGGAAAAGCTCCGAA
GCTTTTAATTTCCAGTGCATCGTTCCTTTATAGCGGGGTCCCAAGCCGCTTTTCGGGTTC
GCGCTCCGGGACCGACTTCACGCTTACGATTTCAAGCCTGCAACCGGAAGATTTCGCCA
CATACTATTGCCAACAGCATTACACGACGCCGCCTACCTTCGGGCAAGGCACGAAGGTG
GAAATCAAACGCGGGGGAGGTGGCTCCGGAGGTGGGGGTTCAGGCGGCGGAGGGTCT
TCGGGTGGAGGGAGTGAGGTTCAGCTTGTGGAATCAGGTGGAGGTTTAGTGCAACCTG
GTGGTAGTTTACGCCTGTCCTGCGCAGCTAGTGGATTCAATATCAAAGACACTTATATCC
ATTGGGTACGTCAAGCCCCTGGGAAAGGACTGGAATGGGTCGCCCGTATTTACCCCACT
AACGGTTATACTCGTTACGCCGACTCTGTTAAGGGACGCTTCACCATTAGTGCGGACAC
ATCTAAAAACACAGCTTACTTGCAGATGAACTCCCTTCGTGCAGAGGACACCGCCGTCT
ACTACTGTAGCCGTTGGGGAGGGGATGGATTTTATGCGATGGACTACTGGGGGCAGGG
CACTCTTGTCACGGTCTCCAGCGGTGGATCAGGCGGAAGTGGCGGTTCAGGTGGGAGT
GGTGGCAGCTTGCAAAGAATGAAACAACTTGAAGACAAGGTTGAAGAATTGCTTTCGAA
AAATTATCACTTGGAAAATGAGGTTGCCAGATTAAAGAAATTAGTTGGCGAACGC

v. Trastuzumab scFv with disulfide-forming Cys residues converted to Ser. Modified codons are shown in red.

GACATTCAGATGACGCAGTCGCCATCAAGCTTAAGCGCCAGTGTGGGTGATCGCGTCA
CAATCACATCCCGTGCTTCCCAAGATGTAAATACCGCGGTGGCCTGGTATCAGCAAAAA
CCGGGAAAAGCTCCGAAGCTTTTAATTTACAGTGCATCGTTCCTTTATAGCGGGGTCCC
AAGCCGCTTTTCGGGTTCGCGCTCCGGGACCGACTTCACGCTTACGATTTCAAGCCTGC
AACCGGAAGATTTCGCCACATACTATTCCCAACAGCATTACACGACGCCGCCTACCTTC
GGGCAAGGCACGAAGGTGGAAATCAAACGCGGGGGAGGTGGCTCCGGAGGTGGGGGT
TCAGGCGGCGGAGGGTCTTCGGGTGGAGGGAGTGAGGTTCAGCTTGTGGAATCAGGT
GGAGGTTTAGTGCAACCTGGTGGTAGTTTACGCCTGTCCTCAGCAGCTAGTGGATTCAA
TATCAAAGACACTTATATCCATTGGGTACGTCAAGCCCCTGGGAAAGGACTGGAATGGG
TCGCCCGTATTTACCCCACTAACGGTTATACTCGTTACGCCGACTCTGTTAAGGGACGC
TTCACCATTAGTGCGGACACATCTAAAAACACAGCTTACTTGCAGATGAACTCCCTTCGT
GCAGAGGACACCGCCGTCTACTACTCTAGCCGTTGGGGAGGGGATGGATTTTATGCGA
TGGACTACTGGGGGCAGGGCACTCTTGTCACGGTCTCCAGC

w. H98 mimetic peptide.

CTTCTGGGGCCATACGAATTATGGGAATTAAGTCAC

x. 5X GGS linker. Used to link YibK or scFv to C-terminal GCN4, SH2 or YibK.

GGTGGATCAGGCGGAAGTGGCGGTTCAGGTGGGAGTGGTGGCAGC

Supplementary References

1 Lim, K. *et al.* Structure of the YibK methyltransferase from Haemophilus influenzae (HI0766): a cofactor bound at a site formed by a knot. *Proteins* **51**, 56-67, doi:10.1002/prot.10323 (2003).
2 Cho, H. S. *et al.* Structure of the extracellular region of HER2 alone and in complex with the Herceptin Fab. *Nature* **421**, 756-760, doi:10.1038/nature01392 (2003).
3 Jiang, B. *et al.* A novel peptide isolated from a phage display peptide library with trastuzumab can mimic antigen epitope of HER-2. *The Journal of biological chemistry* **280**, 4656-4662, doi:10.1074/jbc.M411047200 (2005).
4 Ringquist, S. *et al.* Translation initiation in Escherichia coli: sequences within the ribosome-binding site. *Molecular microbiology* **6**, 1219-1229 (1992).
5 Davis, J. H., Rubin, A. J. & Sauer, R. T. Design, construction and characterization of a set of insulated bacterial promoters. *Nucleic Acids Res* **39**, 1131-1141, doi:10.1093/nar/gkq810 (2011).
6 Esvelt, K. M., Carlson, J. C. & Liu, D. R. A system for the continuous directed evolution of biomolecules. *Nature* **472**, 499-503, doi:10.1038/nature09929 (2011).
7 Hubbard, B. P. *et al.* Continuous directed evolution of DNA-binding proteins to improve TALEN specificity. *Nat Methods* **12**, 939-942, doi:10.1038/nmeth.3515 (2015).
8 Badran, A. H. & Liu, D. R. Development of potent in vivo mutagenesis plasmids with broad mutational spectra. *Nat Commun* **6**, 8425, doi:10.1038/ncomms9425 (2015).
9 Wang, T., Badran, A. H., Huang, T. P. & Liu, D. R. Continuous directed evolution of proteins with improved soluble expression. *Nature Chemical Biology*, doi:10.1038/s41589-018-0121-5 (2018).
10 Schofield, D. A. *et al.* Development of a thermally regulated broad-spectrum promoter system for use in pathogenic gram-positive species. *Applied and environmental microbiology* **69**, 3385-3392, doi:10.1128/AEM.69.6.3385-3392.2003 (2003).
11 Chang, H.-J. *et al.* A Modular Receptor Platform To Expand the Sensing Repertoire of Bacteria. *ACS Synthetic Biology* **7**, 166-175, doi:10.1021/acssynbio.7b00266 (2018).
12 Bolanos-Garcia, V. M. & Davies, O. R. Structural analysis and classification of native proteins from E. coli commonly co-purified by immobilised metal affinity chromatography. *Biochim Biophys Acta* **1760**, 1304-1313, doi:10.1016/j.bbagen.2006.03.027 (2006).
13 Robichon, C., Luo, J., Causey, T. B., Benner, J. S. & Samuelson, J. C. Engineering Escherichia coli BL21(DE3) Derivative Strains To Minimize E. coli Protein Contamination after Purification by Immobilized Metal Affinity Chromatography. *Applied and Environmental Microbiology* **77**, 4634-4646, doi:doi:10.1128/AEM.00119-11 (2011).
14 Schlundt, A. *et al.* Structure-function analysis of the DNA-binding domain of a transmembrane transcriptional activator. *Sci Rep* **7**, 1051, doi:10.1038/s41598-017-01031-9 (2017).
