# Supplementary material for: Disulfide-compatible phage-assisted continuous evolution in the periplasmic space
Source: Nat Commun. 2021 Oct 13;12:5959. doi: 10.1038/s41467-021-26279-8 (PMC8514426; doi:10.1038/s41467-021-26279-8)
Supplement: Supplementary file 2 — Reporting Summary [file 41467_2021_26279_MOESM2_ESM.pdf]

nature portfolio

Corresponding author(s): David R. Liu

Last updated by author(s): Sep 24, 2021

Reporting Summary

Nature Portfolio wishes to improve the reproducibility of the work that we publish. This form provides structure for consistency and transparency in reporting. For further information on Nature Portfolio policies, see our Editorial Policies and the Editorial Policy Checklist.

Statistics

For all statistical analyses, confirm that the following items are present in the figure legend, table legend, main text, or Methods section.

n/a	Confirmed	
☐	☒	The exact sample size (*n*) for each experimental group/condition, given as a discrete number and unit of measurement
☐	☒	A statement on whether measurements were taken from distinct samples or whether the same sample was measured repeatedly
☐	☒	The statistical test(s) used AND whether they are one- or two-sided<br>*Only common tests should be described solely by name; describe more complex techniques in the Methods section.*
☒	☐	A description of all covariates tested
☐	☒	A description of any assumptions or corrections, such as tests of normality and adjustment for multiple comparisons
☐	☒	A full description of the statistical parameters including central tendency (e.g. means) or other basic estimates (e.g. regression coefficient) AND variation (e.g. standard deviation) or associated estimates of uncertainty (e.g. confidence intervals)
☒	☐	For null hypothesis testing, the test statistic (e.g. *F*, *t*, *r*) with confidence intervals, effect sizes, degrees of freedom and *P* value noted<br>*Give P values as exact values whenever suitable.*
☒	☐	For Bayesian analysis, information on the choice of priors and Markov chain Monte Carlo settings
☒	☐	For hierarchical and complex designs, identification of the appropriate level for tests and full reporting of outcomes
☒	☐	Estimates of effect sizes (e.g. Cohen's *d*, Pearson's *r*), indicating how they were calculated

Our web collection on statistics for biologists contains articles on many of the points above.

Software and code

Policy information about availability of computer code

Data collection	None used
Data analysis	ImageJ v 1.50i, Prism 8, MO.Control, MO.Affinity Analysis v3.2

For manuscripts utilizing custom algorithms or software that are central to the research but not yet described in published literature, software must be made available to editors and reviewers. We strongly encourage code deposition in a community repository (e.g. GitHub). See the Nature Portfolio guidelines for submitting code & software for further information.

Data

Policy information about availability of data

All manuscripts must include a data availability statement. This statement should provide the following information, where applicable:

- Accession codes, unique identifiers, or web links for publicly available datasets
- A description of any restrictions on data availability
- For clinical datasets or third party data, please ensure that the statement adheres to our policy

Materials such as plasmids and maps will be made available upon reasonable request. Cited crystal structure data are available in the Protein Database under entry IDS 1J85 and 1N8Z. No large datasets were generated. All other source data can be accessed in the source data document. No code was utilized in this manuscript.

Field-specific reporting

Please select the one below that is the best fit for your research. If you are not sure, read the appropriate sections before making your selection.

- [x] Life sciences
- [ ] Behavioural & social sciences
- [ ] Ecological, evolutionary & environmental sciences

For a reference copy of the document with all sections, see nature.com/documents/nr-reporting-summary-flat.pdf

Life sciences study design

All studies must disclose on these points even when the disclosure is negative.

Sample size	Sample sizes were determined based on literature precedence for directed evolution. Replication is not standard in directed evolution, as the role of stochasticity is such that evolutionary trajectories are not expected to be repeatable. For experiments verifying elements of selection design, 2-4 replicates were used in most cases, with 2 replicates being considered the minimum for phage-based experiments in which values are expected to differ by multiple orders of magnitude between treatments and replicates are performed on separate days. Two replicates was also considered an acceptable minimum for pellet fraction expression assays (western and coomassie gels), as no major claims are made with regard to expression levels in the pellet. For important data such as ELISA, four replicates were conducted where possible. For precedents, see Wang et al. 2018 (https://doi.org/10.1038/s41589-018-0121-5) and Badran et al. 2016 (10.1038/nature17938).
Data exclusions	No data excluded. One data point was removed from experiment S1 (see Source Data) due to pipetting error, but data was not collected for this point. One data point was removed from MST analysis (Fig. S14, supplementary Table 5) due to fluorophore adsorption, but the raw value is provided.
Replication	With the exception of certain evolutions (where replication is not standard in the field as evolutionary trajectories are not expected to be fully repeatable) and the plaque assay shown in Main text Fig. 4C and Supplementary Fig. 8A-D, which was conducted as a visual example, all experiments were replicated at least once. All attempts at replication were successful.
Randomization	Bacteria used for these experiments were grown under identical conditions, so randomization was not used.
Blinding	Bacteria used for these experiments were grown under identical conditions, so blinding was not used.

Reporting for specific materials, systems and methods

We require information from authors about some types of materials, experimental systems and methods used in many studies. Here, indicate whether each material, system or method listed is relevant to your study. If you are not sure if a list item applies to your research, read the appropriate section before selecting a response.

Materials & experimental systems

n/a	Involved in the study	
[ ]	[x]	Antibodies
[x]	[ ]	Eukaryotic cell lines
[x]	[ ]	Palaeontology and archaeology
[x]	[ ]	Animals and other organisms
[x]	[ ]	Human research participants
[x]	[ ]	Clinical data
[x]	[ ]	Dual use research of concern

Methods

n/a	Involved in the study	
[x]	[ ]	ChIP-seq
[x]	[ ]	Flow cytometry
[x]	[ ]	MRI-based neuroimaging

Antibodies

Antibodies used	Antibodies were used for western blotting analysis. The following antibodies were used:<br>Anti-6xHis, mouse monoclonal, abcam ab18184 (http://www.abcam.com/6x-his-tag-antibody-hish8-ab18184.html) lot# GR3349062-1, clone HIS.H8, 1:2000 dilution<br>Anti-GroEL, rabbit polyclonal, Sigma-Aldrich G6532 (https://www.sigmaaldrich.com/catalog/product/sigma/g6532) lot# 0000093479, 1:20,000 dilution<br>Anti-Maltose Binding Protein, mouse monoclonal [R29.6], abcam 65 (https://www.abcam.com/maltose-binding-protein-antibody-r296-ab65.html), lot #GR106061-5, 1:5000 dilution<br>Anti-c-myc antibody, mouse monoclonal (clone ABL-148, ascites fluid), Sigma-Aldrich A5844 (https://www.sigmaaldrich.com/US/en/product/sigma/a5844), lot #038M4859V, 1:2000 dilution<br>Anti-mouse 680RD secondary, goat polyclonal, LI-COR 926-68070 lot# C70427-05, 1:5000 diluton<br>Anti-rabbit 800CW secondary, donkey polyclonal, LI-COR 926-32213 lot# C50512-05, 1:5000 dilution<br>Anti-6X His epitope tag (mouse) monoclonal CY3 conjugated antibody, Rockland 200-304-382 (https://rockland-inc.com/Product.aspx?id=45468), lot # 34680, reconstituted to 5nM in TBS.
Validation	Anti-6xHis, mouse monoclonal, abcam ab18184: validated for western blotting by manufacturer (blotting against rRab27a-His in

whole cell lysate)
Anti-GroEL, rabbit polyclonal, Sigma-Aldrich G6532: validated for western blotting by manufacturer (blotting against GroEL in E. coli bacterial extract)
Anti-c-myc antibody, mouse monoclonal (clone ABL-148, ascites fluid), Sigma-Aldrich A5844: validated for western blotting by manufacturer (blotting against human and mouse c-ABL)
Anti-Maltose Binding Protein, mouse monoclonal [R29.6], abcam 65: validated for western blotting by manufacturer (blotting against native MBP in whole cell lysate)
Anti-mouse 680RD secondary, goat polyclonal, LI-COR 926-68070: validated for western blotting by manufacturer (dot blot against serum proteins)
Anti-rabbit 800CW secondary, donkey polyclonal, LI-COR 926-32213 lot# C50512-05, 1:5000 dilution; validated for western blotting by manufacturer (dot blot against serum proteins)
Anti-6X His epitope tag (mouse) monoclonal CY3 conjugated antibody, Rockland 200-304-382: validated for ELISA and western blotting by manufacturer (blotting and ELISA against recombinant protein)
